# 2D Oxides for Electronics and Optoelectronics

**DOI:** 10.1002/smsc.202200008

**Published:** 2022-06-22

**Authors:** Xiaozong Hu, Kailang Liu, Yongqing Cai, Shuang-Quan Zang, Tianyou Zhai

**Affiliations:** ^1^ Henan Key Laboratory of Crystalline Molecular Functional Materials Henan International Joint Laboratory of Tumor Theranostical Cluster Materials Green Catalysis Center, and College of Chemistry Zhengzhou University Zhengzhou 450001 P. R. China; ^2^ State Key Laboratory of Materials Processing and Die and Mould Technology School of Materials Science and Engineering Huazhong University of Science and Technology Wuhan 430074 P. R. China; ^3^ Joint Key Laboratory of the Ministry of Education Institute of Applied Physics and Materials Engineering University of Macau Taipa 999078 Macau P. R. China

**Keywords:** field-effect transistors, high-κ dielectrics, oxides, photodetectors, 2D materials

## Abstract

In recent years, 2D oxides have attracted considerable attention due to their novel physical properties and excellent stability. With the efforts of researchers, significant progress has been made in the synthesis and electronics and optoelectronics application of 2D oxides. Herein, a systematic review focusing on the preparation of 2D oxides and their applications in electronics and optoelectronics is provided. First, 2D oxides are summarized and classified according to their elements. Then, common preparation methods to synthesize 2D oxides including exfoliation, liquid‐phase synthesis, vapor deposition, surface oxidation of metal, and so on are introduced. Further, the applications of 2D oxides in electronics and optoelectronics are presented. Finally, the current challenges and envisioned development of 2D oxides are commented and prospected.

## Graphical abstract

Due to the excellent properties and air stability, 2D oxides show promising application prospects in optoelectronics and electronics, which are expected to promote the development of the semiconductor industry together with common other 2D materials. Herein, the preparation and application in optoelectronics and electronics of 2D oxides in recent years are summarized.

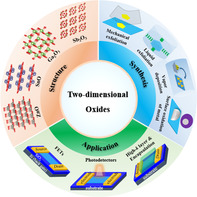

## 1 Introduction

The discovery of graphene opened the door to the world of 2D materials.^[^ ^1^ ^]^ Due to the quantum confinement effect in the direction of the atomic layer, 2D materials exhibit rich and excellent properties.^[^ ^2^, ^3^, ^4^, ^5^, ^6^ ^]^ Especially in the fields of electronics and optoelectronics, 2D materials such as graphene,^[^ ^1^, ^2^ ^]^ transition metal dichalcogenides (TMDs),^[^ ^7^, ^8^, ^9^ ^]^ and black phosphorus (BP).^[^ ^10^, ^11^ ^]^ show promising application potential. The devices, such as field‐effect transistors (FETs), light‐emitting diodes, and photodetectors based on these materials, have been demonstrated to possess superior performance or novel function. Hence, 2D materials are considered as competitive candidates for the next‐generation electronic and optoelectronic materials.

As a group of 2D materials, 2D oxides, which are formed by the O element with a metallic or quasimetallic element, have been gradually attracting increasing attention. Since O can be combined with different elements or at different stoichometric ratios, 2D oxides have relatively rich material species, for example, 2D ZnO,^[^ ^12^ ^]^ SnO,^[^ ^13^ ^]^ MnO_2_,^[^ ^14^ ^]^ MoO_2_,^[^ ^15^ ^]^ MoO_3_,^[^ ^16^ ^]^ and so on. Compared with the typical 2D materials such as graphene, TMDs, and BP, 2D oxides have the following advantages: first, good air stability. As a p‐type semiconductor with high mobility, the application of BP is severely limited by poor air stability. Fortunately, most 2D oxides exhibit excellent air stability attributed to the participation of O, and the elements contained in the materials exist in relatively stable valence states. For example, 2D β‐TeO_2_ reported by Zavabeti et al. exhibits high hole mobility of over 6000 cm^2^ V^−1^ at −50 °C and is expected to become an important competitor of BP. Second advantage is the low requirement of synthetic environment. The preparation of most 2D materials requires a high synthetic environment, such as the synthesis of TMDs requires isolation of oxygen and the preparation of 2D BP in a glove box. For 2D oxides, they generally have low requirements for synthetic environment and even in the atmospheric environment can realize their synthesis. For example, 2D β‐Ga_2_O_3_, β‐TeO_2_, Bi_2_O_3_, and so on can be prepared in atmospheric environment by naturally oxidizing liquid metals. Third advantage is the excellent ultraviolet (UV) detection performance. Most of the reported oxides have a wide bandgap and their light absorption is in the UV region. 2D ZnO and β‐Ga_2_O_3_ for UV detection have been reported. Therefore, 2D oxides can make up the shortcoming of conventional 2D materials in the detection of UV region.

In recent years, 2D oxides have been studied and shown great potential for electronics and optoelectronics. For example, the photodetectors based on ZnO nanosheets showed excellent performance for ultraviolet detection: the highest responsivity reached 2.0 × 10^4^ A W^−1^ and the detectivity is as high as 6.83 × 10^14^ Jones under 254 nm light.^[^ ^17^ ^]^ 2D β‐Ga_2_O_3_‐based photodetectors showed high selectivity to light wavelength: the photocurrent declines sharply when the incident wavelength exceeds 354 nm.^[^ ^18^ ^]^ Xiong's group reported photodetectors based on Fe_3_O_4_ nanosheets with a detection range from 375 nm to 10.6 μm.^[^ ^19^ ^]^ Some 2D oxides showed outstanding performance in FETs. The hole mobility of FETs based on few‐layer hexagonal TiO_2_ reached 950 cm^2^ V^−1^ s^−1^ at room temperature.^[^ ^20^ ^]^ β‐TeO_2_‐based FETs exhibited a high switching on/off ratio of 10^6^ and low subthreshold swing of 103 ± 3 mV dec^−1^. Moreover, an exciting thing is that the hole mobility of the device is over 6000 cm^2^ V^−1^ s^−1^ when the temperature cooled down to −50 °C, which is comparable with BP, and it possess much better air stability.^[^ ^21^ ^]^ Recently, we proposed the concept of 2D inorganic molecular crystals and realized the preparation of a wafer‐scale van der Waals (vdW) high‐κ dielectric of 2D Sb_2_O_3_ layer.^[^ ^22^ ^]^ The MoS_2_/Sb_2_O_3_ FETs exhibited an ultrahigh on/off ratio of 10^8^, enhanced carrier mobility of 145 cm^2^ V^−1^ s^−1^ (compared with 26 cm^2^ V^−1^ s^−1^ for the same device on SiO_2_), and ideal subthreshold swing of 64 mV dec^−1^. In addition, 2D oxides have also shown application potential in piezoelectric transistors,^[^ ^23^ ^]^ artificial synaptic devices,^[^ ^24^, ^25^ ^]^ memristors,^[^ ^26^ ^]^ and anisotropic detection.^[^ ^27^, ^28^ ^]^

At present, more 2D oxides have been reported, the synthesis methods are emerging constantly, and their application in the electronics and optoelectronics is increasingly extensive. Given the great advance that has been made in the studies of 2D oxides, a review is needed to summarize its advance and provide an outlook to its future development. In this article, we will first classify the reported 2D oxides according to the elemental type. Then we introduce the common preparation methods to synthesize 2D oxides including mechanical exfoliation, liquid‐phase synthesis, vapor deposition, and surface oxidation of metal (SOM). Further, the application of 2D oxides in electronics and optoelectronics is presented in detail. Finally, the current challenges and envisioned development of 2D oxides are commented and prospected upon.

## 2 Classification, Typical Structures, and Properties of 2D Oxides

### 2.1 Classification

**Table** **1** summarizes some basic information of reported 2D oxides including material structure (layered or nonlayered) and preparation methods. Moreover, according to the elements, we classified the reported 2D oxides into three categories (summarized in Table 1): 1) transition metal oxides^[^ ^29^ ^]^ (TMOs) including MoO_3_,^[^ ^30^, ^31^, ^32^ ^]^ MoO_2_,^[^ ^33^, ^34^ ^]^ WO_3_,^[^ ^35^, ^36^ ^]^ V_2_O_5_,^[^ ^37^ ^]^ and Cr_2_O_3_;^[^ ^38^ ^]^ 2) main‐group metal oxides (MMOs), such as Ga_2_O_3_,^[^ ^39^, ^40^ ^]^ In_2_O_3_,^[^ ^41^ ^]^ PbO,^[^ ^42^, ^43^ ^]^ and Bi_2_O_3_; and^[^ ^44^ ^]^ 3) semimetallic oxides (SMOS), such as h‐GeO_2_ and^[^ ^20^ ^]^ β‐TeO_2_.^[^ ^21^ ^]^

**Table 1:** Summary of the reported 2D oxides in experiments

Type	Materials	Structure	Methods	References
TMOs	ZnO	Nonlayered	AILE,a) SOM,b) MBEc)	[12, 24, 175]
	MoO_2_	Nonlayered	CVDd)	[15]
	MnO_2_	Layered	LPEe)	[14, 176]
	VO_2_	Nonlayered	CVD	[28]
	TiO_2_	Nonlayered	SOM	[177]
	h‐TiO_2_	Layered	SOM	[20]
	RuO_2_	Layered	LPE	[97]
	HfO_2_	Nonlayered	SOM	[122]
	Gd_2_O_3_	Nonlayered	SOM	[122]
	Cr_2_O_3_	Layered	CVD	[38]
	Y_2_O_3_	Nonlayered	MBE	[113]
	h‐Fe_2_O_3_	Layered	SOM	[20]
	α‐MoO_3_	Layered	CVD, ME,f)	[30, 173]
	WO_3_	Nonlayered	ME and Annealing	[21]
	Fe_3_O_4_	Nonlayered	CVD	[19]
	V_2_O_5_	Layered	LPE	[178]
	TiO_x_	Layered	LPE	[80]
MMOs	MgO	Nonlayered	Conformal anneal synthesis	[130]
	SnO	Layered	SOM, PLDg), LPE	[47, 179, 180]
	PbO	Layered	SOM, LPE	[43, 51, 89, 90]
	In_2_O_3_	Nonlayered	SOM	[41]
	Al_2_O_3_	Nonlayered	SOM	[122]
	β‐Ga_2_O_3_	Nonlayered	SOM	[124, 181]
	α‐Bi_2_O_3_ γ‐Bi_2_O_3_	Nonlayered	CVD, SOM	[44, 117]
SMOs	Sb_2_O_3_	Layered	CVD, Thermal deposition	[22, 53]
	β‐TeO_2_	Layered	SOM	[21]
	h‐GeO_2_	Layered	SOM	[20]

a) AILE adaptive ionic layer epitaxy;

b) SOM: surface oxidation of metal;

c) MBE: molecular beam epitaxy;

d) CVD: chemical vapor deposition;

e) LPE: liquid‐phase exfoliation;

f) ME: mechanical exfoliation;

g) PLD: pulsed laser deposition.

### 2.2 Structures of Typical 2D Oxides

**Figure 1:**
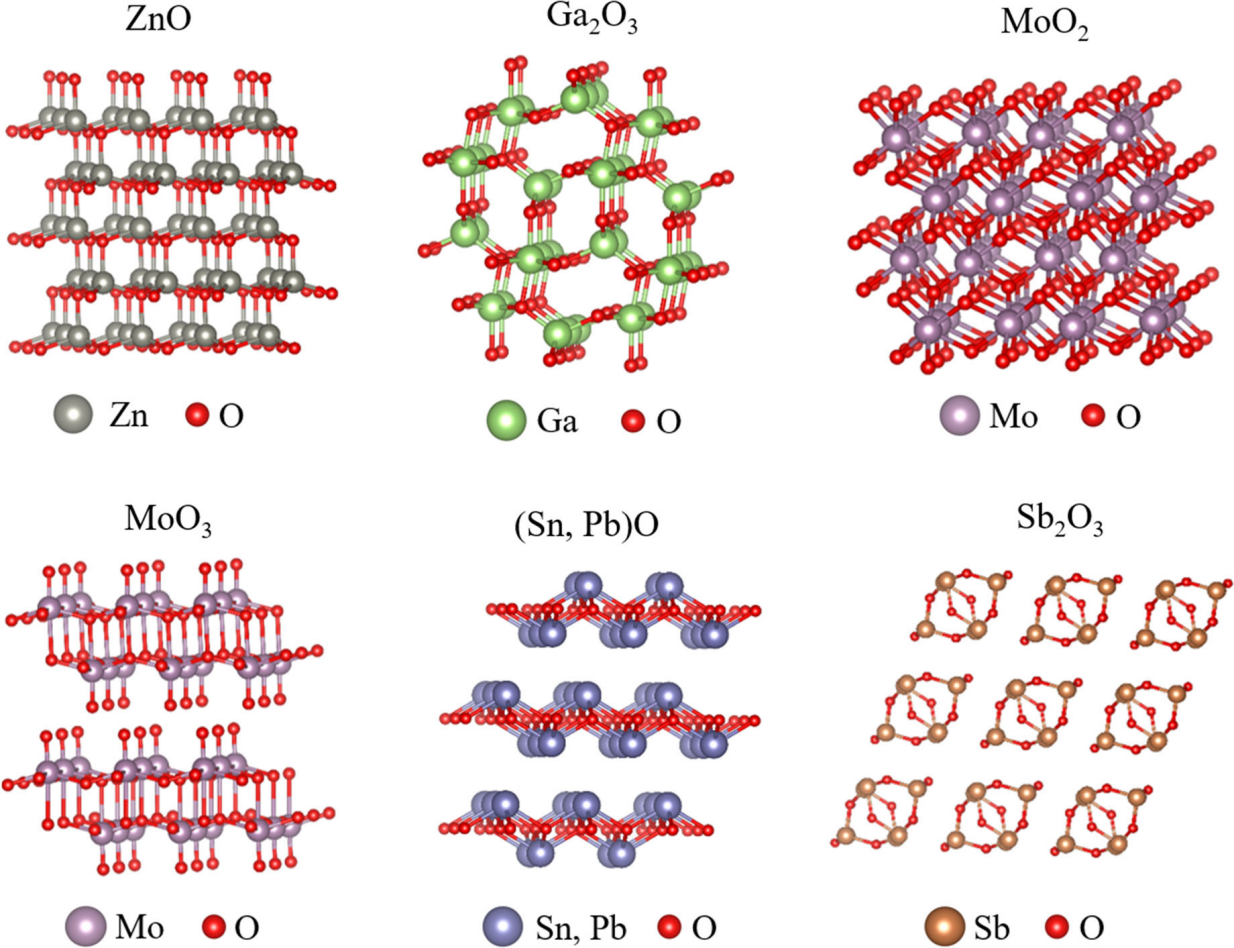
Some common and typical structures of 2D oxides.

The crystal structures of some common and typical 2D oxides are shown in **Figure** **1**. We noticed that the reported 2D oxides contain a variety of structures, such as nonlayered ZnO,^[^ ^45^ ^]^ Ga_2_O_3_,^[^ ^46^ ^]^ and MoO_2_,^[^ ^15^ ^]^ whose atoms are connected by strong chemical bonds in all directions and thus, refrain from a straightforward synthesis of the 2D form. Layered 2D oxides like SnO,^[^ ^47^, ^48^ ^]^ α‐MoO_3_,^[^ ^49^, ^50^ ^]^ and PbO^[^ ^51^, ^52^ ^]^ have structures like MoS_2_, in which the atoms are connected by strong chemical bonds within layers and the layers stack together via weak van der Waals (vdW) forces. Sb_2_O_3_ is an inorganic molecular crystal,^[^ ^53^ ^]^ which consists of 0D inorganic molecules of Sb_4_O_6_. Within Sb_4_O_6_, each Sb atom is connected with three O atoms, and two Sb atoms, to form an adamantanoid cage. The adamantanoid cages are connected by vdW forces. The research on this kind of materials is quite popular due to their special structure.^[^ ^22^, ^54^, ^55^ ^]^

### 2.3 Basic Optical and Electrical Properties of Typical 2D Oxides

To better explore the application of emerging materials, it is necessary to know their basic properties.^[^ ^56^, ^57^, ^58^ ^]^ In this section, we will mainly introduce the basic optical and electrical properties of typical 2D oxides.

#### 2.3.1 Optical Properties

**Figure 2:**
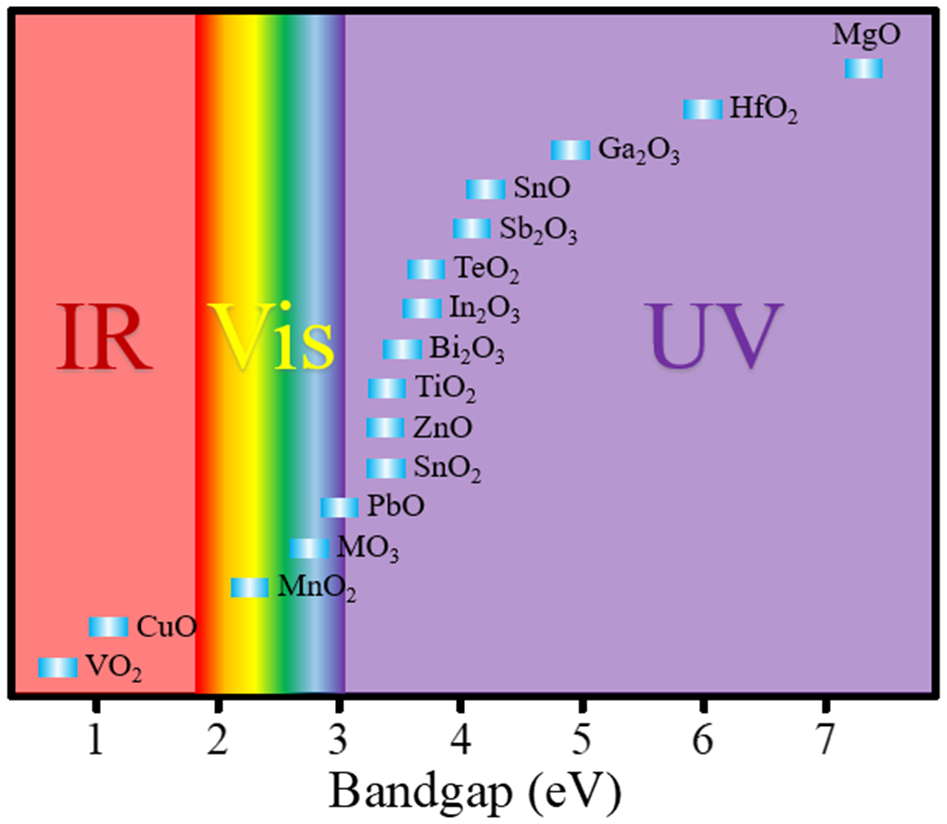
The bandgap of reported 2D Oxides.

**Figure 3:**
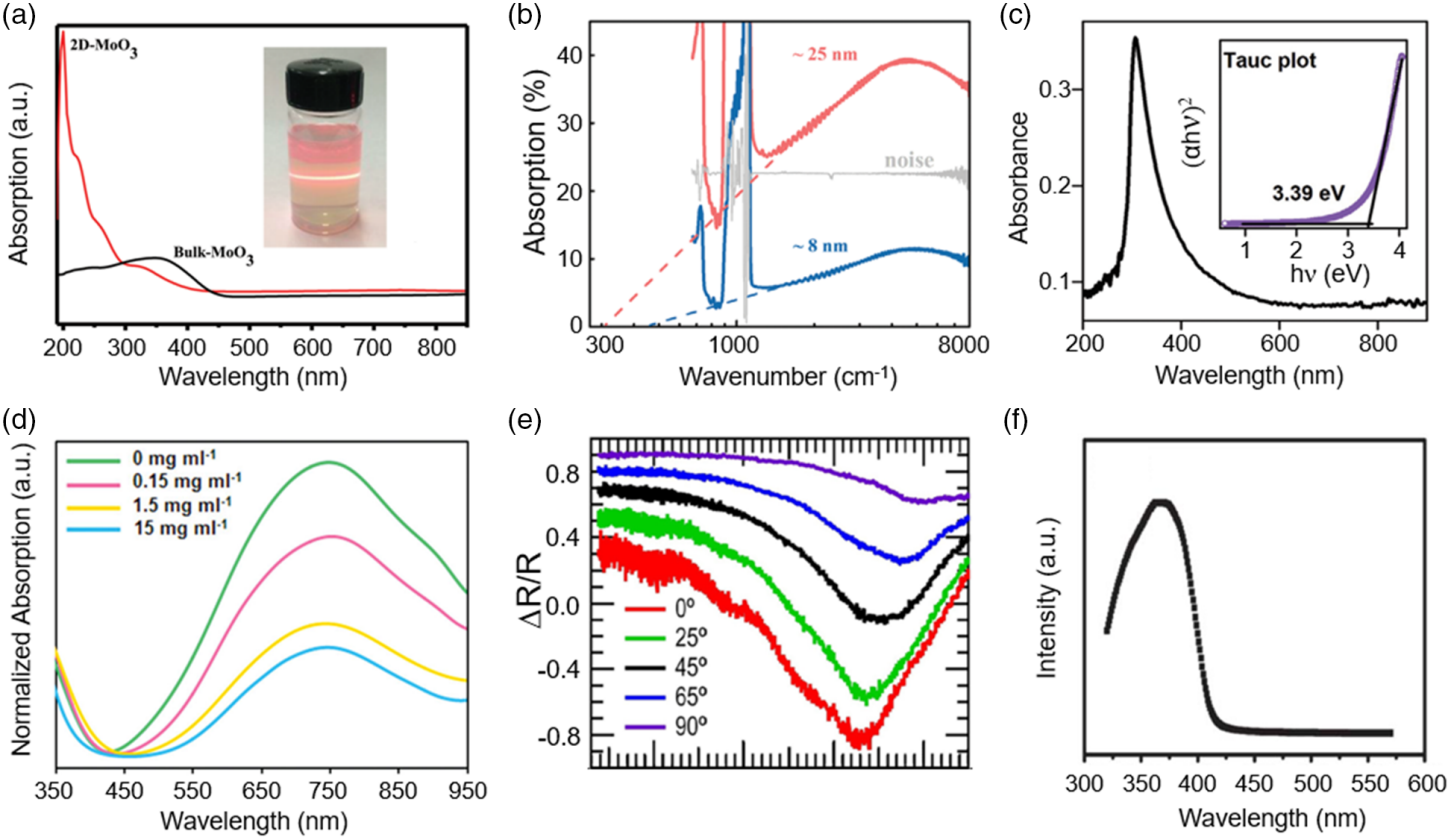
The optical properties of typical 2D Oxides. a) The UV–vis absorption spectra of bulk MoO_3_ and exfoliated 2D MoO_3_ suspension. Reproduced with permission.^[^ ^161^ ^]^ Copyright 2016, The Royal Society of Chemistry. b) Infrared absorption spectra of Fe_3_O_4_ nanosheets synthesized by CVD. Reproduced with permission.^[^ ^19^ ^]^ Copyright 2019, Wiley‐VCH. c) The UV–vis–NIR spectra of ZnO nanosheets synthesized by liquid‐metal exfoliation. Inset: Tauc plot extracted from the absorption spectrum which reveals a direct bandgap of 3.39 eV. Reproduced with permission.^[^ ^24^ ^]^ Copyright 2021, Wiley‐VCH. d) Normalized absorption spectra of 2D MoO_3_ nanoshets with different BSA concentration. Reproduced with permission.^[^ ^49^ ^]^ Copyright 2014, Wiley‐VCH. e) The linear reflectance contrast spectra (*ΔR/R*) of MoO_3_ under different incident angles. Reproduced with permission.^[^ ^62^ ^]^ Copyright 2021, AIP Publishing. f) PL specture of β‐Ga_2_O_3_ nanosheets. Reproduced with permission.^[^ ^18^ ^]^ Copyright 2014, The Royal Society of Chemistry.

**Figure** **2** presents the bandgap of synthesized 2D oxides that cover a wide light spectrum from the infrared to vacuum UV. We also can notice that most 2D oxides have a wide bandgap. These mean that 2D oxide‐based photodetectors have the potential to achieve a wide detection range and perform well in the UV region. Due to the quantum size effect, 2D oxides exhibit different optical properties from their bulk materials. As shown in **Figure** **3**a, the optical absorption of 2D MoO_3_ shows strong blueshift compared with bulk MoO_3_. As mentioned earlier, 2D oxides process a wide range of light absorption. The absorption of 2D Fe_3_O_4_ nanosheets is demonstrated and presented in Figure 3b.^[^ ^19^ ^]^ The absorption edges of Fe_3_O_4_ with thicknesses of 8 and 25 nm are located at 463 and 310 cm^−1^, corresponding to the light wavelength of 21.6 and 32.3 μm, respectively. Figure 3c shows the UV–vis–NIR spectra of the ZnO nanosheets; we can notice a strong absorption in UV region and cutoff point occurs at 450 nm.^[^ ^24^ ^]^ There are other 2D oxides with similar property such as TiO_2_ and^[^ ^59^, ^60^ ^]^ β‐Ga_2_O_3_.^[^ ^61^ ^]^ This property indicates that 2D oxides have the potential for building high‐performance and selective UV photodetectors. In addition, Alsaif et al. demonstrated the applicability of 2D MoO_3_ in optical biosensing.^[^ ^49^ ^]^ The absorption spectra of 2D MoO_3_ with bovine serum albumin (BSA) is shown in Figure 3d; it is found that the absorption peak of 2D MoO_3_ is significantly reduced with the increase in the concentration of BSA. In‐plane anisotropy is an important property of 2D materials. Some 2D oxides also exhibit in‐plane anisotropy, such as MoO_3_,^[^ ^62^ ^]^ β‐TeO_2_,^[^ ^21^, ^63^ ^]^ and β‐Ga_2_O_3_.^[^ ^64^ ^]^ Figure 3e exhibits the optical reflectance spectroscopy of MoO_3_ nanosheet with the different linearly polarized incident light angles.^[^ ^62^ ^]^ The linear reflectance contrast spectra (Δ*R*/*R*) show that the intensity changed with the incident light rotating from parallel to the *b*‐axis to parallel to the *c*‐axis. 2D ZnO and^[^ ^17^ ^]^ β‐Ga_2_O_3_^[^ ^64^ ^]^ have fluorescence (PL) properties (Figure 3f), which can be used in micro–nano light‐emitting devices.

#### 2.3.2 Electrical Properties

**Figure 4:**
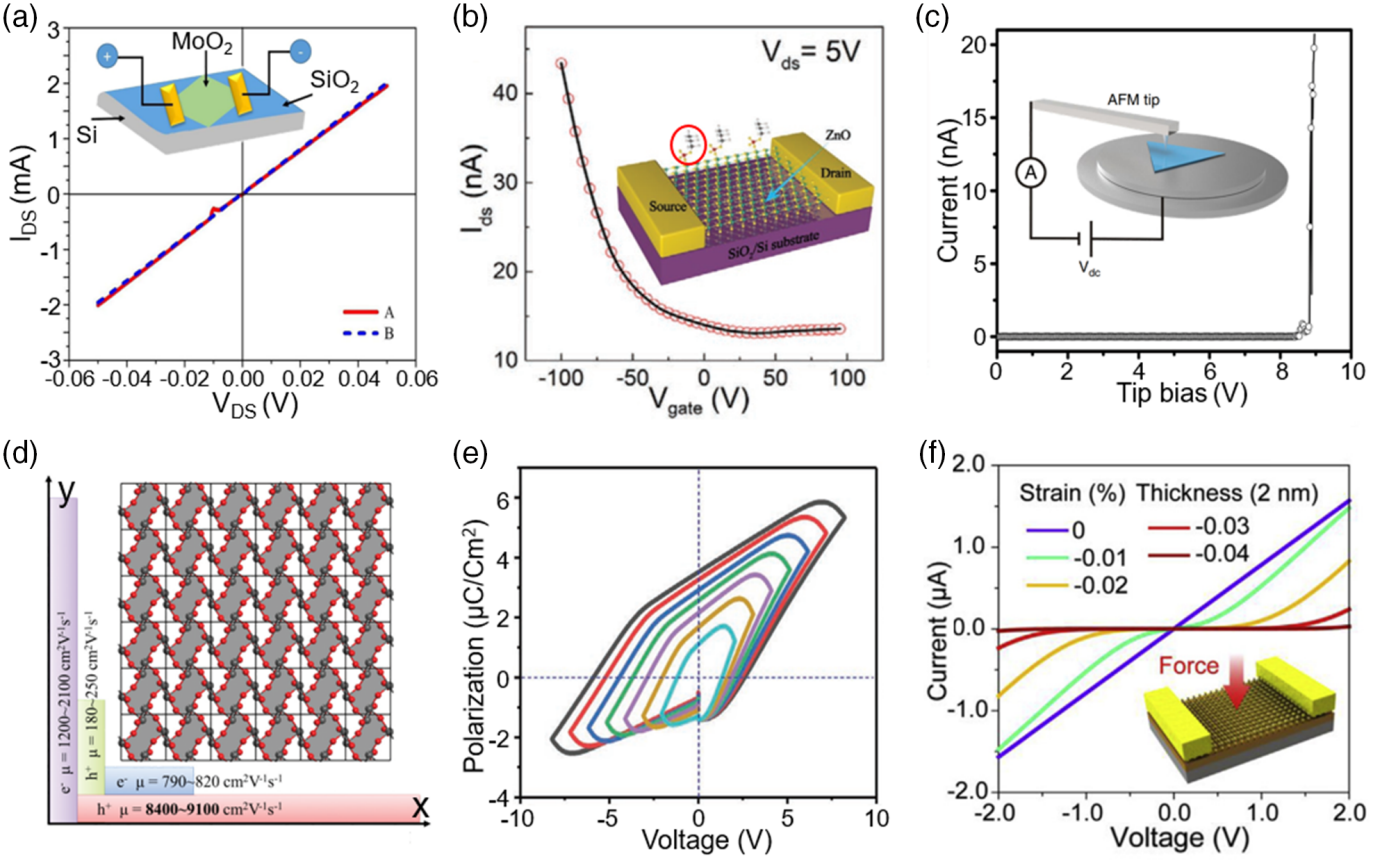
The electrical properties of typical 2D oxides. a) *I–V* curves of device based on MoO_2_ nanosheet. Reproduced under the terms of the CC‐BY 4.0 license.^[^ ^34^ ^]^ Copyright 2020, The Authors, published by Springer Nature. b) Transfer curves of 2D ZnO FET. Reproduced with permission.^[^ ^17^ ^]^ Copyright 2020, Wiley‐VCH. c) Current–voltage curve measured through the ultrathin SbO_1.93_ crystal. Reproduced under the terms of the CC‐BY 4.0 license.^[^ ^55^ ^]^ Copyright 2020, The Authors, published by Springer Nature. d) The hole and electron mobility of monolayer β‐TeO_2_ along the *x* and the *y* directions calculated at 300 K. Reproduced with permission.^[^ ^63^ ^]^ Copyright 2018, The Royal Society of Chemistry. e) The bulk *P*–*V* hysteresis curves with different scanning voltage ranges. Reproduced with permission.^[^ ^68^ ^]^ Copyright 2021, Wiley‐VCH. f) Theoretically calculated *I*_ds_–*V*_ds_ characteristics of 2D ZnO piezotronic devices under a series of compressive strains on the channel. Reproduced with permission.^[^ ^69^ ^]^ Copyright 2019, Elsevier Ltd.

2D oxides cover metals, semiconductors, and insulators. **Figure** **4**a exhibits the *I–V* curves of device based on 2D MoO_2_ nanosheets. 2D MoO shows excellent conductivity with the resistance of only 25 Ω at ±0.05 V.^[^ ^34^ ^]^ The calculated electric structure and electronic density of states show that MoO_2_ exhibits a metallic character. The high‐density free electron in metallic MoO_2_ may provide the possibility for surface plasmon resonance excited by the incident laser. In fact, most 2D oxides are semiconductors. There are some interesting electrical properties of 2D oxides, for example, 2D MoO_3_ prepared by different methods shows different conductivity types and even insulation properties.^[^ ^62^, ^65^, ^66^ ^]^ ZnO is a typical semiconductor and exhibits strong n‐type conductivity due to the native defects. Moreover, there are few successful p‐type doping for ZnO.^[^ ^12^ ^]^ However, Yu et al. synthesized ZnO nanosheets that show p‐type conductivity (Figure 4b). They thought the surface‐adsorbed molecules influence the electrical properties of ZnO due to its ultrathin thickness. This means that the electrical properties of 2D oxides with ultrathin structure can be regulated more easily. Some 2D oxides present good electrical isolation, which can be used as dielectric layers, such as Sb_2_O_3_,^[^ ^53^ ^]^ SbO_1.93_,^[^ ^55^ ^]^ and Y_2_O_3_.^[^ ^67^ ^]^ Yang et al.^[^ ^55^ ^]^ reported a high‐quality ultrathin insulator of 2D SbO_1.93_, which has a high dielectric constant (≈100) and a large breakdown electric filed (≈5.7 GV m^−1^) (Figure 4c). Liu et al^[^ ^22^ ^]^ fabricated wafer‐scale vdW dielectric layers using the inorganic molecular crystal Sb_2_O_3_ with high‐κ. Anisotropy is also reflected in the electrical properties of 2D oxides. As shown in Figure 4 d, monolayer β‐TeO_2_ shows that hole mobility along *x* direction (*b*‐axis) is up to 8400–9100 cm^2^ V^−1^ s^−1^, in contrast to 180–250 cm^2^ V^−1^ s^−1^ along *y* direction (*a*‐axis), due to the quite different effective mass, high elastic modulus, and deformation potential along different direction.^[^ ^63^ ^]^ The result is confirmed experimentally.^[^ ^21^ ^]^ Recently, researchers found that HfO_2_ maintains ferroelectricity even with a thickness of less than 5 nm, which means that it has good application prospects in nonvolatile ferroelectric memory devices.^[^ ^68^ ^]^ Figure 4e shows the bulk *P–V* (polarization vs. applied potential) hysteresis curves of the HfO_2_‐based device with different scanning voltage ranges. A typical hysteresis loop appeared in the *P–V* results and loop opening improved with increasing scanning range. As the noncentrosymmetric structure at thickness of a few atomic layers is maintained, ZnO nanosheets still have piezoelectric properties.^[^ ^23^ ^]^ Figure 4f shows the theoretically calculated *I–V* curves of 2D ZnO piezotronic devices under a series of compressive strains. The currents are suppressed under larger compressive strains.^[^ ^69^ ^]^ This properties can be used to fabricate piezotronic transistors and logic devices.^[^ ^23^ ^]^

## 3 Synthesis Methods

Achieving high‐quality ultrathin 2D oxides is crucial for studying their fundamental properties and practical applications. We can notice from Table 1 that a considerable amount of oxides are of a nonlayered structure, making the preparation of 2D oxides more difficult than layered graphene, TMDs, and BP. However, with the continuous efforts of researchers, a variety of methods for the preparation of ultrathin 2D oxides have been reported,^[^ ^60^, ^70^, ^71^, ^72^, ^73^, ^74^, ^75^, ^76^, ^77^, ^78^, ^79^, ^80^, ^81^ ^]^ mainly including exfoliation, liquid‐phase synthesis (LPS), vapor deposition, and SOM. In this section, we will give a brief overview and comment on the synthesis methods.

### 3.1 Exfoliation

Exfoliation methods reduce the bulk materials to atomic thickness by external force. At present, mechanical exfoliation and liquid‐phase exfoliation (LPE) are commonly used to prepare 2D oxides.

#### 3.1.1 Mechanical Exfoliation

Mechanical exfoliation is an effective method to obtain various ultrathin flakes from their bulk crystal materials.^[^ ^57^, ^82^, ^83^, ^84^ ^]^ The first 2D material, graphene, is prepared by this method. Monolayer‐to‐few‐layer flakes can be obtained by mechanical exfoliation.^[^ ^1^ ^]^ In general, the mechanically exfoliated flakes possess high crystal quality, cleanliness, and intrinsic properties, suitable for the construction of conceptual devices and fundamental properties’ research.

**Figure 5:**
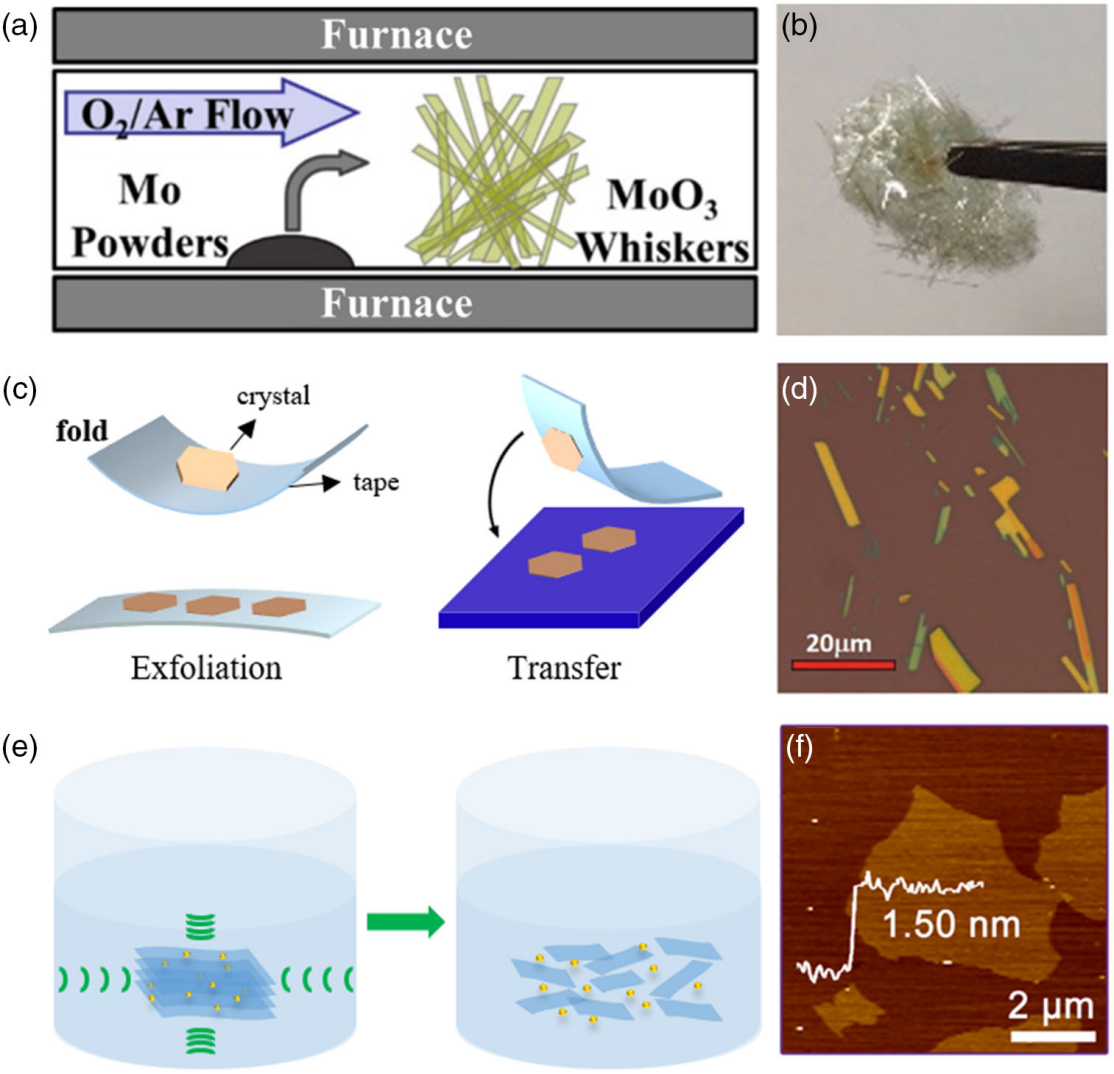
Exfoliation of 2D oxides. a) Schematic illustration of growth of MoO_3_ whiskers. b) OM of MoO_3_ whiskers. a, b) Reproduced with permission.^[^ ^86^ ^]^ Copyright 2019, American Chemical Society. c) Schematic illustration of mechanical exfoliation. d) OM of exfoliated MoO_3_ flakes. Reproduced with permission.^[^ ^30^ ^]^ Copyright 2020, Wiley‐VCH. e) Schematic illustration of LPE. f) AFM of TiO_*x*_ flakes. Reproduced with permission.^[^ ^80^ ^]^ Copyright 2021, American Chemical Society.

Like graphene, ultrathin 2D oxides can also be obtained by exfoliating their high‐quality bulk crystals. While commonly single crystal is prepared by chemical vapor transport (CVT) methods,^[^ ^57^, ^83^, ^85^ ^]^ oxide bulk crystals are usually obtained by chemical vapor deposition (CVD) methods.^[^ ^86^, ^87^ ^]^ Taking MoO_3_, as an example, Razmyar et al.^[^ ^86^ ^]^ used Mo powder as precursor and O_2_ as reactant gas to prepare MoO_3_ single crystal. After reaction at high temperature in a CVD system, MoO_3_ whiskers were obtained in the downstream of quartz tube. The CVD schematic figure and photograph of MoO_3_ whiskers are shown in **Figure** **5**a,b. Figure 5c shows the process of mechanical exfoliation. Bulk crystal with a layered structure is stuck on the scotch tape, and the vdW interaction between layers can be overcome by repeatedly sticking the bulk crystal and thereby peeling off ultrathin 2D flakes. Ultrathin 2D MoO_3_ flakes with the lateral size of about 20 μm obtained by mechanical exfoliation are displayed in Figure 5d.^[^ ^30^ ^]^ From optical micrography (OM), we can notice that the 2D MoO_3_ flakes possess a smooth and clean surface. Furthermore, the flakes obtained by mechanical exfoliation can be transferred to a variety of substrates for the construction of semiconductor devices. However, mechanical exfoliation is only effective for layered materials, and the thickness of obtained flakes demonstrates large randomness. Due to its uncontrollability over thickness, size, and low preparation efficiency, this method is rather suitable for fundamental research than for industrial production.^[^ ^83^, ^84^, ^88^ ^]^

#### 3.1.2 Liquid‐Phase Exfoliation

The LPE is a method that can realize the large‐scale preparation of ultra 2D materials. The raw materials used in LPE are usually single crystal or powder of the target product.^[^ ^89^, ^90^, ^91^, ^92^, ^93^ ^]^ Previous studies have demonstrated that suitable solvents can minimize the energy for the exfoliation of the materials, to facilitate the exfoliation of bulk materials.^[^ ^91^, ^92^ ^]^ The solvents such as acetone, ethanol, DMF (dimethylformamide), NMP (*N*‐methyl‐2pyrrolidone), and DMSO (dimethyl sulfoxide) are usually used. Figure 5e shows the schematic illustration of LPE methods. In order to better obtain 2D materials, sonication and ion intercalation are commonly used to promote the exfoliation process.

So far, a variety of 2D oxides (e.g., MoO_3_,^[^ ^94^ ^]^ WO_3_,^[^ ^95^, ^96^ ^]^ RuO_2_^[^ ^97^ ^]^) have been successfully synthesized by this method. Figure 5f exhibits the atomic force microscope (AFM) images of TiO_*x*_ nanosheets synthesized by LPE.^[^ ^80^ ^]^ In general, layered 2D oxides can be exfoliated in solution with sonication assistance.^[^ ^86^, ^87^ ^]^ However, to further weaken the interlayer interaction of the materials and promote the exfoliation process, small ions or molecules are often inserted into the interspace of layered materials as intercalant agents.^[^ ^98^, ^99^ ^]^ Alsaif et al.^[^ ^94^ ^]^ inserted H^+^ ions into the interspace of MoO_3_ and transformed 2D MoO_3_ into H*x*MoO_3_ due to the H^+^ ions’ bonding with the edge‐shared oxygen and terminal oxygen atoms. Through this method, 2D α‐MoO_3_ nanosheets with only five layers were obtained. In addition, Azam et al.^[^ ^36^ ^]^ reported preparation of 2D nonlayered WO_3_ nanosheets by LPE. First, WS_2_ powder was partially oxidized and exfoliated in NMP with intercalation and sonication assistance to obtain WS_*x*_O_*y*_ nanosheets. Then, the nanosheets were completely transformed into WO_3_ in 4 m HNO_3_ solution. This strategy of exfoliating layered materials and converting them into nonlayered 2D target materials is undoubtedly interesting and worth being extended.

### 3.2 Liquid‐Phase Synthesis

LPS methods are also commonly used to synthesize 2D materials.^[^ ^91^, ^92^, ^100^, ^101^ ^]^ Ultrathin 2D oxides could also be prepared by these methods.^[^ ^17^, ^102^ ^]^ In this section, we summarized several common LPS methods, including adaptive ionic layer epitaxy (AILE), self‐assembly, and salt‐templated epitaxy.

#### 3.2.1 Adaptive Ionic Layer Epitaxy

**Figure 6:**
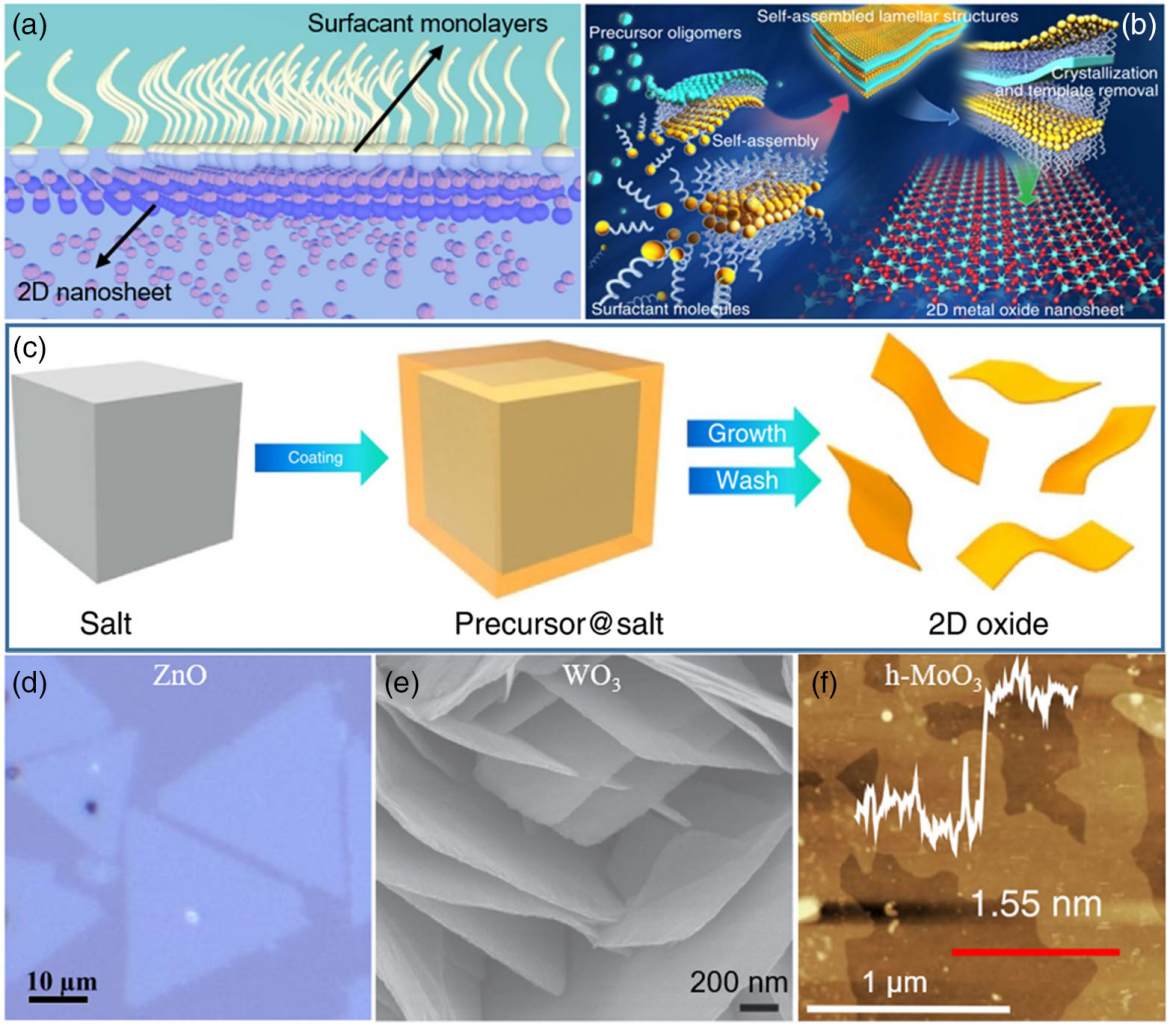
Solution‐phase synthesis methods for the preparation of 2D oxides. a) Schematic illustration of AILE. b) Schematic illustration of self‐assembly. c) Schematic illustration of salt‐templated. d) Optical microscopy image of the 2D ZnO nanosheets. e) SEM image of the 2D WO_3_ nanosheets. f) AFM image of the 2D h‐MoO_3_ nanosheets. a,d) Reproduced under the terms of the CC‐BY 4.0 license.^[^ ^12^ ^]^ Copyright 2016, The Authors, published by Springer Nature. b,e) Reproduced with permission.^[^ ^95^ ^]^ Copyright 2014, Springer Nature. c,f) Reproduced under the terms of the CC‐BY 4.0 license.^[^ ^29^ ^]^ Copyright 2014, The Authors, published by Springer Nature.

The principle of AILE methods is that the surfactants self‐assemble into a monolayer at the solution–air interface, serving as a soft template, and guide the nucleation and epitaxial growth of nanosheets (the schematic illustration is shown in **Figure** **6**a).^[^ ^12^, ^102^, ^103^ ^]^ Wang et al.^[^ ^12^ ^]^ used the sodium oleylsulfate as surfactant and subsequently spread it over the surface of an aqueous solution containing Zn(NO_3_)_2_ and hexamethylenetetramine. Under the oleylsulfate antion monolayers, Zn^2+^ cations are gathered and precipitate into nanosheets. By this method, triangular ZnO nanosheets with thickness of 1–2 nm are obtained at a suitable temperature (Figure 6d). The reason of the long‐range self‐alignment is thought to be the synergy of vdW interaction in the hydrocarbon tails and the strong correlation between the oleylsulfate headgroups and the Zn^2+^ ions below. In addition, during the growth process of 2D nanosheets, the local packing density of the surfactant monolayer would spontaneously adapt to the nanosheets lattice. By choosing a suitable combination of anionic surfactant monolayer and metal ion solution, other 2D oxides such as NiO were synthesized. AILE demonstrated potential in preparing nonlayer 2D oxides.^[^ ^17^, ^102^ ^]^

#### 3.2.2 Self‐Assembly

In recently years, self‐assembly has also been applied to synthesize 2D oxides.^[^ ^70^ ^]^ Figure 6b exhibits the process and mechanism of preparing 2D oxides by self‐assembly. First, surfactant molecules spontaneously formed inverse lamellar micelles. Metal oxide precursor polymerizes and crystallizes into 2D nanosheets with atomic thickness. Then, metal oxide nanosheets and surfactant molecules are strategically and collaboratively self‐assembled into lamellar structures. Finally, 2D oxides can be obtained after removing the surfactant templates (Figure 6e). It is important that this method has excellent scalability, via which 2D nanosheets including TiO_2_, ZnO, Co_3_O_4_, and WO_3_ can be prepared by self‐assembly.^[^ ^95^ ^]^

#### 3.2.3 Salt Templated

The process of salt‐templated method for preparing 2D oxides is shown in Figure 6c.^[^ ^29^ ^]^ The precursor was first coated on the inorganic salt (NaCl, KCl etc.) by mixing the molecular precursor solution and a large volume of salt microcrystals. Next, 2D oxides were produced by drying the solution and further annealing the mixture at high temperature. At last, 2D oxides nanosheets were obtained by washing the mixture to remove the salt.^[^ ^104^ ^]^ Xiao et al.^[^ ^29^ ^]^ synthesized a series of 2D oxides such as h‐MoO_3_, h*‐*WO_3_, MoO_2_, and MnO by this method, and they found that the thickness of the 2D oxides could be controlled by mixing a limited volume of the dilute oxide precursor solution with a large quantity of salt microcrystals. The AFM image of h*‐*MoO_3_ with a thickness of 1.55 nm is shown in Figure 6f. This method can be considered to produce 2D oxides in large quantities since the inorganic salt is cheap and easy to remove.^[^ ^29^ ^]^

Liquid‐phase synthesis has advantages in terms of low‐cost, large‐scale production, and transfer not limited by substrates. However, due to the influence of solvent, the as‐synthesized nanosheets tend to have poor crystallinity and many impurities and defects, which seriously affect the performance of electronic and optoelectronic devices.

### 3.3 Vapor Deposition

The preparation of 2D materials by vapor deposition methods is common.^[^ ^105^ ^]^ So far, vapor deposition methods to prepare 2D oxides mainly include molecular beam epitaxy (MBE)^[^ ^106^, ^107^, ^108^ ^]^ and CVD.^[^ ^19^, ^53^ ^]^

#### 3.3.1 MBE

**Figure 7:**
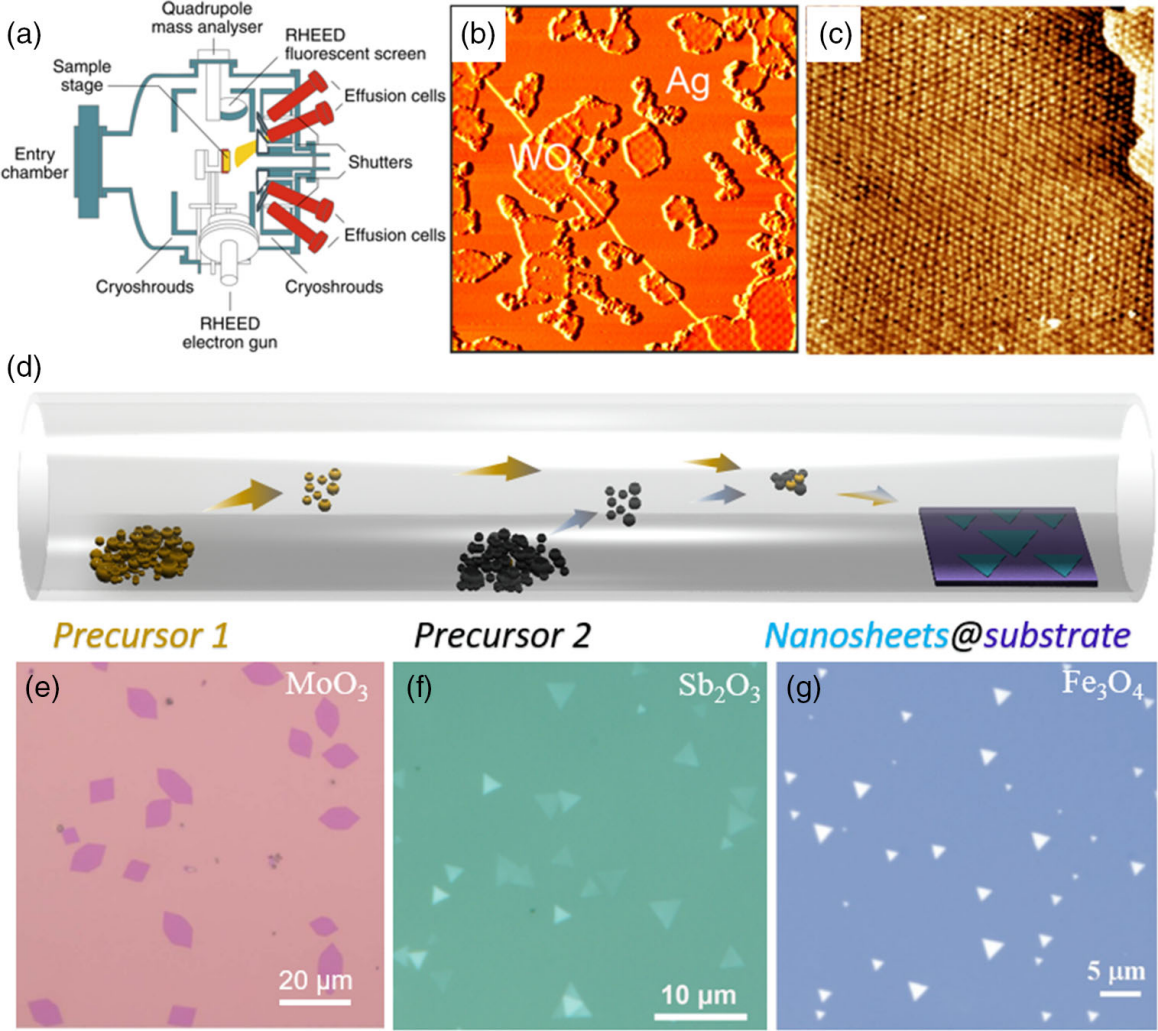
MBE and CVD methods of 2D oxides. a) Schematic illustration of MBE system. Reproduced with permission.^[^ ^109^ ^]^ Copyright 2019, Springer Nature. b) STM images of the WO_3_ layer on Ag(100) scale: 150 × 150 nm^2^. Reproduced with permission.^[^ ^108^ ^]^ Copyright 2019, American Chemical Society. c) STM images of Y_2_O_3_/graphene/Pt(111). Reproduced with permission.^[^ ^113^ ^]^ Copyright 2013, Springer Nature. d) Schematic illustration of the CVD method. Reproduced with permission. OM of 2D oxides synthesized by CVD. e) MoO_3_ nanoplates. Reproduced with permission.^[^ ^173^ ^]^ Copyright 2021, IOP Publishing. f) Sb_2_O_3_ nanosheets. Reproduced under the terms of the CC‐BY 4.0 license.^[^ ^53^ ^]^ Copyright 2019, The Authors, published by Springer Nature. g) Fe_3_O_4_ nanosheets. Reproduced with permission.^[^ ^19^ ^]^ Copyright 2020, Wiley‐VCH GmbH.

MBE is a physical vapor deposition method that is often used to prepare 2D materials (schematic of the MBE system^[^ ^109^ ^]^ is shown in **Figure** **7**a), which could offer large‐sized, high‐quality single crystal nanoflakes.^[^ ^83^, ^105^, ^110^ ^]^ By adjusting the deposition rate of elements, the thickness and stoichiometry of 2D nanoflakes can be highly controlled. With this method, Negreiros et al.^[^ ^108^ ^]^ prepared a fully stoichiometric and epitaxially ordered 2D WO_3_ layer on Ag(100) surface. The scanning tunneling microscopy (STM) image of the 2D WO_3_ layer on Ag(100) substrate is shown in Figure 7b: small WO_3_ islands coexist with bare Ag areas. Furthermore, a well‐ordered and atomically flat WO_3_ layer can be obtained by further deposition. Though there is a large lattice mismatch of 7.6% between WO_3_ layer and Ag(100) surface, the nearly defect‐free and large‐area WO_3_ layer is formed, which means that the layer resembles a “freestanding” 2D oxide layer and weakly couples to Ag(100). Integrating the high‐*k* dielectric layer on 2D materials is the key to realizing the application of 2D devices.^[^ ^111^, ^112^ ^]^ Addou and co‐workers^[^ ^113^ ^]^ grew the high‐*k* dielectric, Y_2_O_3_, on Pt‐supported graphene by MBE. A uniform monolayer Y_2_O_3_ was deposited on graphene/Pt(111) in ultrahigh vacuum at room temperature. Ordered structures appear after annealing at above 550 °C (as shown in the Figure 7c). The results of X‐ray photoemission spectroscopy measurements showed a shift of the Fermi level in graphene after depositing Y_2_O_3_, which means that the Y_2_O_3_ layers could realize charge doping of metal‐supported graphene.^[^ ^113^ ^]^ MBE shows advantages in the preparation of 2D oxides with high‐quality and controllable thickness, but its practical application is limited by harsh growth conditions and low growth efficiency.

#### 3.3.2 CVD

CVD is considered as the most promising method for preparing 2D materials,^[^ ^7^, ^8^, ^114^ ^]^ which can realize the preparation of high‐quality 2D materials with controllable thickness and excellent reproducibility in a large scale.^[^ ^7^, ^115^, ^116^ ^]^ The schematic illustration of CVD is shown in Figure 7 d. The precursors are placed in a quartz tube and evaporated to gas phase under high temperature. With the transport by carrier gas, the chemical reaction takes place to grow the target product, which is deposited on the preselected substrates. This is a complicated process in which, the precursor types, carrier gas, temperature, and substrates will affect the process of chemical reaction and the growth of samples. At present, CVD has been widely used to prepare 2D oxides.^[^ ^15^, ^28^, ^33^, ^34^, ^117^, ^118^ ^]^ Guo et al. used MoO_3_ mixed with a small amount of NaCl as precursor, reacting with S powder at high temperatures. 2D MoS_2_, MoO_3_–MoS_2_ heterojunction, and MoO_3_ nanosheets can be obtained when the powder is in different zones, respectively. For layered materials, the growth of 2D nanosheets by CVD is relatively easy to achieve, but for nonlayered materials, the CVD process needs to be modified. Recently, Zhai's group^[^ ^53^ ^]^ designed a passivator‐assisted CVD and realized the preparation of the ultrathin 2D Sb_2_O_3_ inorganic molecular crystal (Figure 7f). Different from the 2D layered materials, Sb_2_O_3_ consists of 0D molecules Sb_4_O_6_. Previous report has proven that 0D molecules tend to spontaneously assemble to 1D rods instead of 2D flakes due to the absence of orientation preference.^[^ ^119^ ^]^ InCl_3_ or Se was used as the passivator for promoting the growth of high‐energy planes and suppressing the growth of low‐energy planes, leading to the formation of 2D Sb_2_O_3_. Novel structures are likely to lead to novel physical properties, so 2D inorganic molecules may open up opportunities for future electronics and optoelectronics devices. Compared with the mechanical exfoliation method, the CVD method could not only realize the preparation of layered materials, but also the nonlayered materials. Xiong's group^[^ ^19^ ^]^ reported the air‐stable nonlayered ultrathin Fe_3_O_4_ nanosheets prepared by the space‐confined CVD method, by constructing the sandwich structure of mica/oxide Fe foil/mica forming a unique confined space for sample growth. In the process of CVD, BrI_3_ was used as the surface passivator to suppress unsaturated dangling bonds. Great progress has been made in synthesizing 2D oxides by CVD. However, so far no wafer‐scale 2D oxides have been reported. So there is still a long way to go toward the large‐scale growth of ultrathin 2D oxides by CVD.

### 3.4 Surface Oxidation of Metal

**Figure 8:**
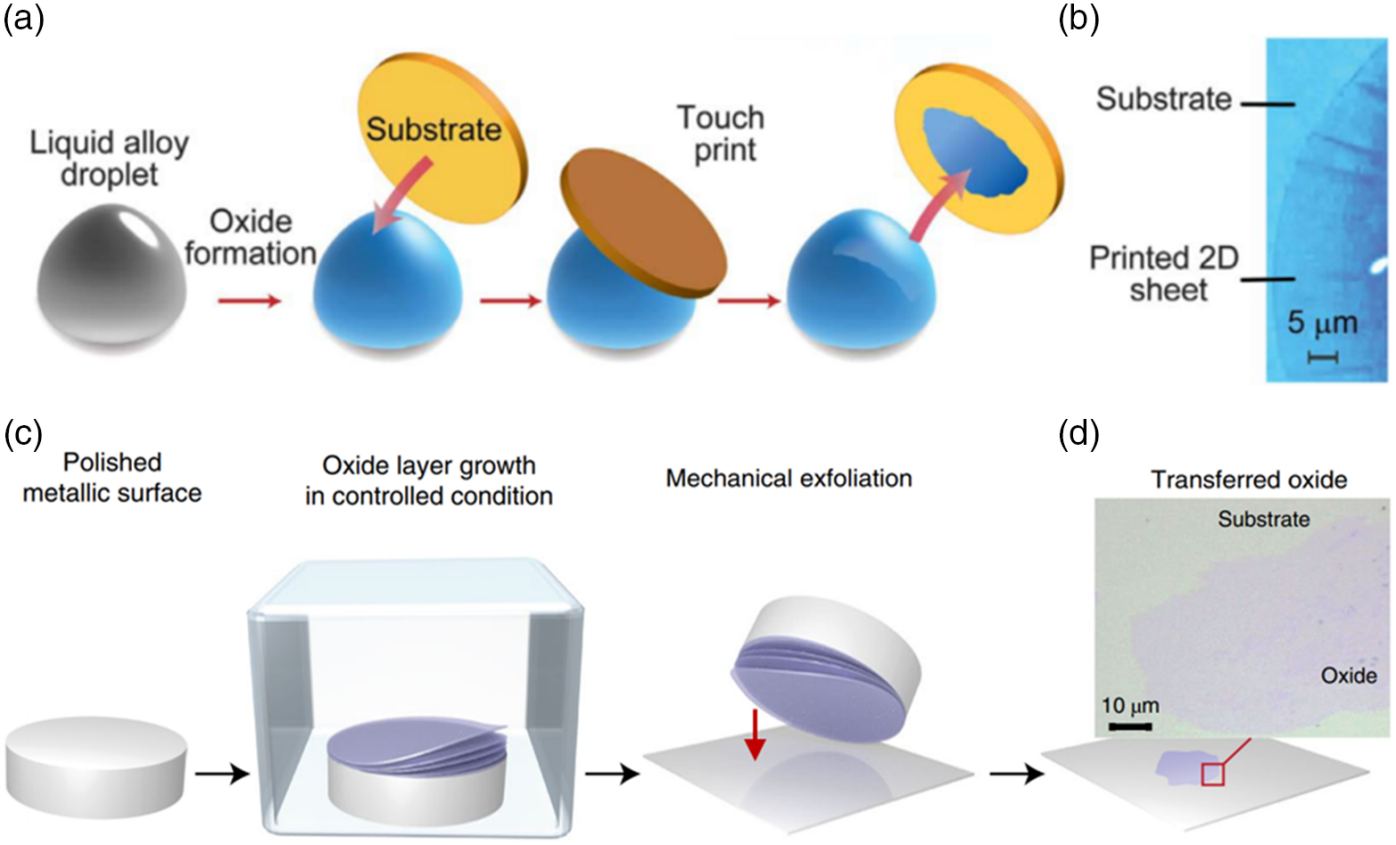
SOM methods of 2D oxides. a) Schematic representation of 2D oxides prepared by the SOLM and transferred to the substrate by van der Waals exfoliation. b) A typical optical image of the 2D sheet on the substrate. a,b) Reproduced with permission.^[^ ^122^ ^]^ Copyright 2017, American Association for the Advancement of Science. c) Schematic of the surface oxidation of solid metal and stamping the metal surface onto a substrate for transferring 2D oxide layer. d) A typical optical image of the transferred 2D oxide layer. c, d) Reproduced with permission.^[^ ^20^ ^]^ Copyright 2021, Springer Nature.

SOM is a new method to prepare ultrathin 2D oxides. For most metals, the self‐limiting thin oxide layer forms easily at the metal–air interface. Based on this principle, a method for preparing ultrathin 2D oxides was developed by stripping the oxide skin from the metal.^[^ ^120^ ^]^ When the oxide skin formed on the surface of liquid metal, the interaction force between the liquid metal and the 2D oxide skin was weak and localized due to the nonpolar nature of liquid metal.^[^ ^121^ ^]^ As a result, it is possible to separate the 2D oxide skin from the liquid metal. The process of preparing 2D oxides by surface oxidation of liquid metal (SOLM) is shown in **Figure** **8**a. So far, a large number of ultrathin 2D oxides were synthesized by this method, such as Al_2_O_3_,^[^ ^122^ ^]^ Bi_2_O_3_,^[^ ^44^ ^]^ SnO,^[^ ^47^, ^123^ ^]^ Ga_2_O_3_,^[^ ^124^, ^125^, ^126^ ^]^ Cu_*x*_O,^[^ ^127^ ^]^ even high‐melting‐point metal oxide HfO_2_,^[^ ^122^ ^]^ and heterojunction.^[^ ^41^ ^]^ The reported SOLM method is roughly the same in principle, with slight difference in detail. Zavabeti et al.^[^ ^122^ ^]^ added less than 1 wt% Hf, Al, and Gd in the Galinstan alloy to prepare ultrathin 2D HfO_2_, Al_2_O_3_, and Gd_2_O_3_. The oxide skin is transferred onto the substrate by substrate touching and separating the surface of the liquid. Further, Alsaif et al.^[^ ^41^ ^]^ constructed 2D p‐SnO/n‐In_2_O_3_ heterostructure by transferring oxide layers of liquid metal surfaces in two steps. First, pure tin metal was melted at 300 °C inside a glove box with oxygen concentration of 10–100 ppm. SnO layer was formed on the surface of the liquid tin and then transferred to the preheated SiO_2_/Si substrates. Second, In_2_O_3_ layer was obtained by similar protocol and transferred on the SnO layer to build a p‐SnO/n‐In_2_O_3_ heterostructure. In addition, compressed air is injected into the liquid metal covered by deionized water; high‐yield suspensions of the target oxide nanosheets can be obtained due to the rapid reaction of oxygen with the surface of liquid metals and the exfoliation of the oxide skin via the explosion of gas bubbles.^[^ ^121^, ^122^ ^]^

The SOLM is suitable for the preparation of 2D oxides of metals with low melting point. However, for those metals with high melting point, it is necessary to form alloys to reduce the melting point, and the preparation process becomes more complicated. Recently, Zhang and co‐workers^[^ ^128^ ^]^ reported a method for preparing 2D metal oxides by oxidation of solid metal surfaces. The process is shown in Figure 8c: the polished bulk metal is placed in an oxygen‐deficient environment which can slow down the oxygen penetration into the metal lattices, together with a suitable temperature to support the formation of uniform oxide layers.^[^ ^20^ ^]^ The oxide layers can be easily transferred by stamping them onto the target substrate on account of being strongly bound within the plane but weakly bound to the metal surface.^[^ ^128^ ^]^ This strategy has been proven effective for the preparation of a variety of 2D oxides, for example, TMOs (TiO_2_, Fe_2_O_3_, Ni_2_O_3_), MMOs (Al_2_O_3_), and lanthanide oxide (Gd_2_O_3_) and SMOs (GeO_2_).^[^ ^20^ ^]^

With the current development trend, the SOM method could realize the preparation of a variety of 2D oxides with high quality and large scale. However, there are still some limitations. For example, the 2D oxide films obtained by this method are usually incomplete in a large area, which is hard to be used in practical application. Compared with the samples prepared by CVD or mechanical exfoliation, their crystallinity is poor.

### 3.5 Other Methods

**Figure 9:**
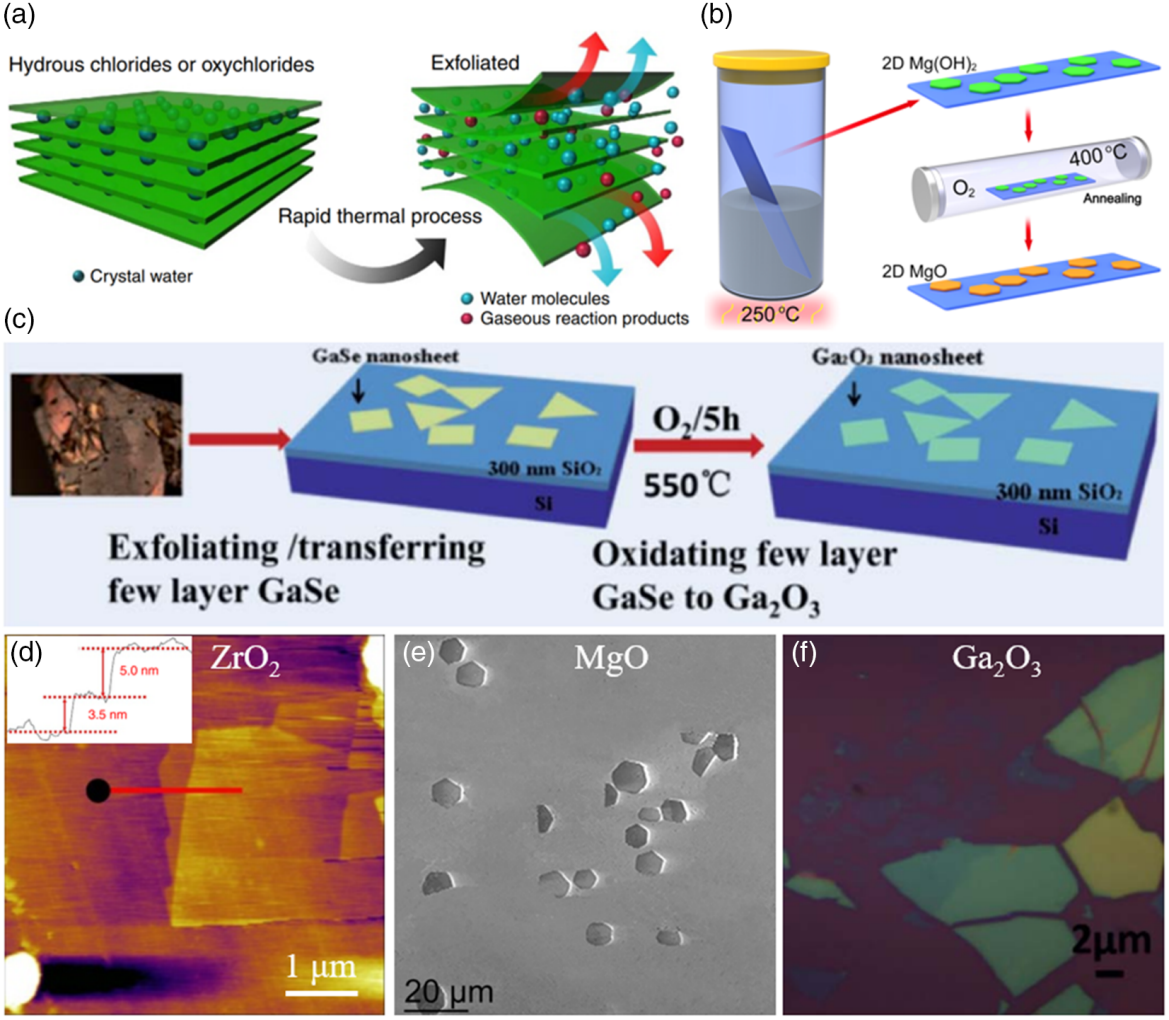
Indirect methods for the preparation of 2D oxides. a) Schematic of the exfoliation of hydrous chlorides or oxychlorides by rapid thermal annealing process. b) Schematic of the CAS process for preparing 2D MgO. c) Schematic of the process for preparing 2D MgO. d) AFM images of 2D ZrO_2_ nanosheets. e) SEM images of 2D MgO nanosheets. f) OM of 2D Ga_2_O_3_ nanosheets. a,d) Reproduced under the terms of the CC‐BY 4.0 license.^[^ ^129^ ^]^ Copyright 2016, The Authors, published by Springer Nature. b,e) Reproduced with permission.^[^ ^130^ ^]^ Copyright 2018, American Chemical Society. c,f) Reproduced with permission.^[^ ^18^ ^]^ Copyright 2014, The Royal Society of Chemistry.

In this section, we will introduce several uncommon but meaningful methods for the preparation of 2D oxides. Some nonlayered oxides cannot be prepared by direct exfoliation methods. Zhao and co‐workers^[^ ^129^ ^]^ reported a method for the fabrication of 2D oxide nanosheets by rapid thermal annealing of their corresponding hydrous–chloride compounds which possess layered structures, as shown in **Figure** **9**a. During the process of rapid thermal annealing, large quantities of steam or other gaseous reaction products are released within a short time, which can generate high pressure. The force induced by gas generation would drive the exfoliation of layered hydrous chlorides or oxychlorides and the as‐exfoliated nanosheets further react spontaneously to form 2D oxides. In such a way, 2D oxides such as Cr_2_O_3_, ZrO_2_, Al_2_O_3_, and Y_2_O_3_ can be obtained by rapid thermal annealing of CrCl_3_·6H_2_O, ZrOCl_2_·8H_2_O, AlCl_3_·6H_2_O, and YCl_3_·6H_2_O, respectively. Similarly, Zheng et al.^[^ ^130^ ^]^ reported a conformal anneal synthesis (CAS) method to prepare 2D MgO. As shown in Figure 9b, layered Mg(OH)_2_ nanosheets grew on the sapphire substrate first; then the Mg(OH)_2_ nanosheets precursor was annealed in a closed furnace with oxygen. High‐quality 2D MgO nanosheets were obtained finally. Figure 9e exhibits the typical scanning electron microscopy (SEM) images of 2D MgO nanosheets with a size of 3–15 μm. Figure 9c shows the synthesis of 2D β‐Ga_2_O_3_ by the direct oxidation of GaSe nanosheets.^[^ ^18^ ^]^ GaSe nanosheets can be obtained easily by mechanically exfoliating bulk GaSe crystal due to its layered structure. After oxidation of ultrathin GaSe nanosheets at high temperature, nonlayered β‐Ga_2_O_3_ was obtained (Figure 9f). In all of such methods, ultrathin nanosheets of layered compounds corresponding to the target 2D oxides are prepared first, and then the target 2D oxides are obtained by further treatment. Although these methods are not common at present, they have guiding significance for the preparation of nonlayered 2D oxides.

## 4 Applications

With the development of research, the application of 2D oxides in optoelectronics and electronics has been explored. In this section, we will highlight and comment on representative studies about 2D oxides for the application mainly including FETs, photodetectors, high‐*k* dielectric layer, and sensors.

### 4.1 Field‐Effect Transistors

Through the efforts of the researchers, the FETs based on 2D materials have made great advances. However, there are still some problems that need to be solved. For example, the FETs based on TMDs always show low mobility and the lack of air‐stable and high‐mobility p‐type FETs. To some extent, 2D oxides can make up for the shortages.

**Figure 10:**
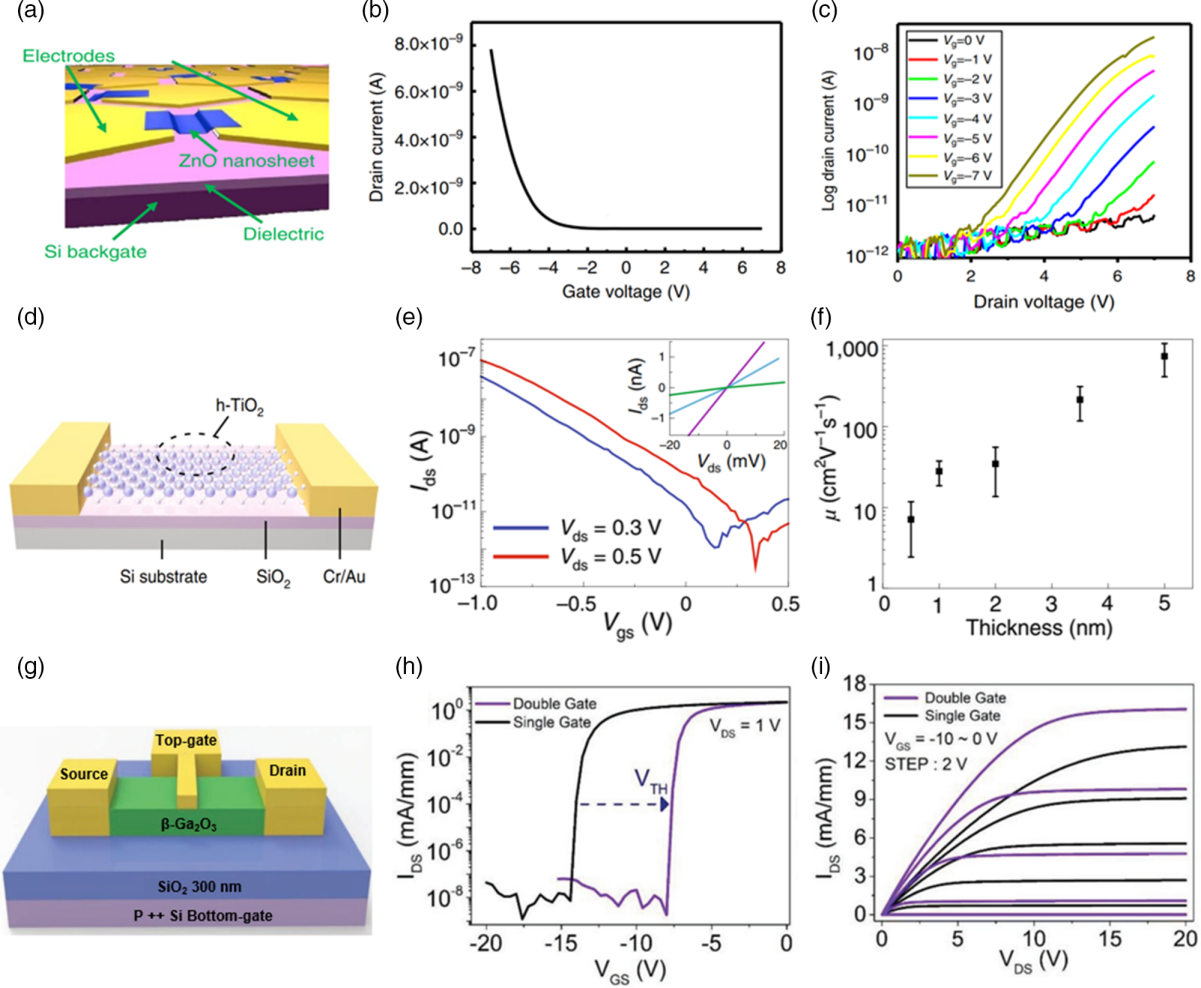
FETs based on 2D oxides. a) Schematic diagram of ZnO FETs. b) The transfer characteristic curve of FETs based on ZnO nanosheets. c) The output characteristic curve of ZnO FETs at different gate voltages. a–c) Reproduced under the terms of the CC‐BY 4.0 license.^[^ ^12^ ^]^ Copyright 2016, The Authors, published by Springer Nature. d) Schematic diagram of FETs based on h‐TiO_2_. e) The transfer characteristic curve of the h‐TiO_2_ FETs with *V*_ds_ being 0.3 and 0.5 V. Inset: *I–V* cruves with a linear behavior. f) The mobility of h‐TiO_2_ FETs as a function with thickness of 2D h‐TiO_2_. d–f) Reproduced with permission.^[^ ^20^ ^]^ Copyright 2021, Springer Nature. g) Schematic illustration of the double‐gate β‐Ga_2_O_3_ FETs. h) Transfer and j) output curves of single top‐gate in comparison with double‐gate β‐Ga_2_O_3_ FETs. g–j) Reproduced with permission.^[^ ^133^ ^]^ Copyright 2019, Wiley‐VCH.

At present, there are many 2D oxides that have been used for FETs, such as SnO,^[^ ^47^ ^]^ SnO_2_,^[^ ^131^ ^]^ MoO_3_,^[^ ^65^, ^66^ ^]^ Ga_2_O_3_,^[^ ^132^ ^]^ TiO_2_, and^[^ ^20^ ^]^ β‐TeO_2_.^[^ ^21^ ^]^ **Figure** **10**a shows the structure diagram of the FETs based on ZnO nanosheets, which are transferred to a prefabricated electrode. ZnO typically shows n‐type conductivity due to native defects. However, the transfer curve of the ZnO nanosheets showed p‐type conductivity: the source–drain current decreased gradually with the gate voltage sweeping from negative to positive. The output curves further confirmed the result.^[^ ^12^ ^]^ Recently, Zhang et al.^[^ ^20^ ^]^ prepared h‐TiO_2_ by SOM and built back‐gate FETs based on the as‐grown nanosheets (Figure 10d). From the transfer curves (Figure 10e), we noticed that the FETs showed p‐type behavior. h‐TiO_2_ nanosheets exhibited strong thickness‐dependent hole mobility. While the field‐effect mobility was 10 cm^2^ V^−1^ s^−1^ for a 0.5 nm‐thick device and reached 950 cm^2^ V^−1^ s^−1^ for the 5 nm‐thick device at room temperature (Figure 10f), Zavabeti et al.^[^ ^21^ ^]^ constructed FETs based on β‐TeO_2_ nanosheets, and the FETs showed p‐type switching with a high on/off ratio of 10^6^ and small subthreshold swing of 130 ± 3 mV dec^−1^. When the temperature cooled down to −50 °C, the hole mobility could reach 6000 cm^2^ V^−1^ s^−1^. In addition, double‐gate field‐effect modulating could realize better control of channel material carriers.^[^ ^133^ ^]^ Asymmetric double‐gate β‐Ga_2_O_3_ FET is shown in Figure 10 g. The device consists of top‐gate metal–semiconductor FET and back‐gate metal‐oxide FET. Figure 10h,i presents the transfer and output curves of asymmetric double‐gate FET compared with top‐gate FET. Under the regulation and control of double gate, the threshold voltage shifts toward the positive direction of about 6.1 V, and the device shows lower on‐resistance. This is beneficial to reducing the power consumption of devices. The carrier mobility reached 184 cm^2^ V^−1^ s^−1^, higher than single back‐gate or top‐gate β‐Ga_2_O_3_ FETs.^[^ ^133^ ^]^

Furthermore, we summarize the reported FETs based on the 2D oxides and show them in **Table** **2**. We notice some of the p‐type 2D oxide FETs, such as SnO, ZnO, and α‐MoO_3_, which means that the 2D oxides can complement the deficiencies of 2D common materials in p‐type FETs. However, the current research on 2D oxide FETs is not enough; most of the FETs show poor performance, such as low mobility and on/off ratio.

**Table 2:** Summary of FETs based on 2D oxides

Nature [‐type]	Materials	Method	Mobility [cm^2^ V^−1^ s^−1^]	On/off ratio	References
p	h‐TiO_2_	SOMa)	950	10^4^	[20]
p	β‐TeO_2_	SOM	232 6000 (223 K)	10^6^	[21]
p	SnO	SOM	0.7	20	[47]
p	SnO	Pulsed laser deposition	1.9	–	[179]
p	β‐Ga_2_O_3_	SOM	21	7 × 10^4^	[182]
p	ZnO	AILEb)	0.10	–	[12]
p	α‐MoO_3_	LPEc)	600	>10^5^	[94]
p	α‐MoO_3_	MEd)	>1100	<10^3^	[65]
n	α‐MoO_3_	Epitaxial growth	0.03	10^3^	[66]
n	α‐MoO_3_	Chemical thinning	175	4000	[183]
n	α‐MoO_3_	Vapor–solid process	*μ*_b_: 0.09e) *μ*_c_: 0.04f)	–	[27]
–	Ga_2_O_3_/MoS_2_	SOM	–	–	[132]

a) SOM: surface oxidation of metal;

b) AILE: adaptive ionic layer epitaxy;

c) LPE: liquid‐phase exfoliation;

d) ME: mechanical exfoliation;

e) *μ*_b_: mobility along *b*‐axis;

f) *μ*_c_: mobility along *c*‐axis.

### 4.2 Photodetectors

Photodetectors are promising applications for 2D oxides. So far, a variety of 2D oxides have been reported for the application of photodetectors due to their unique properties.^[^ ^19^, ^38^, ^130^, ^134^, ^135^ ^]^

**Figure 11:**
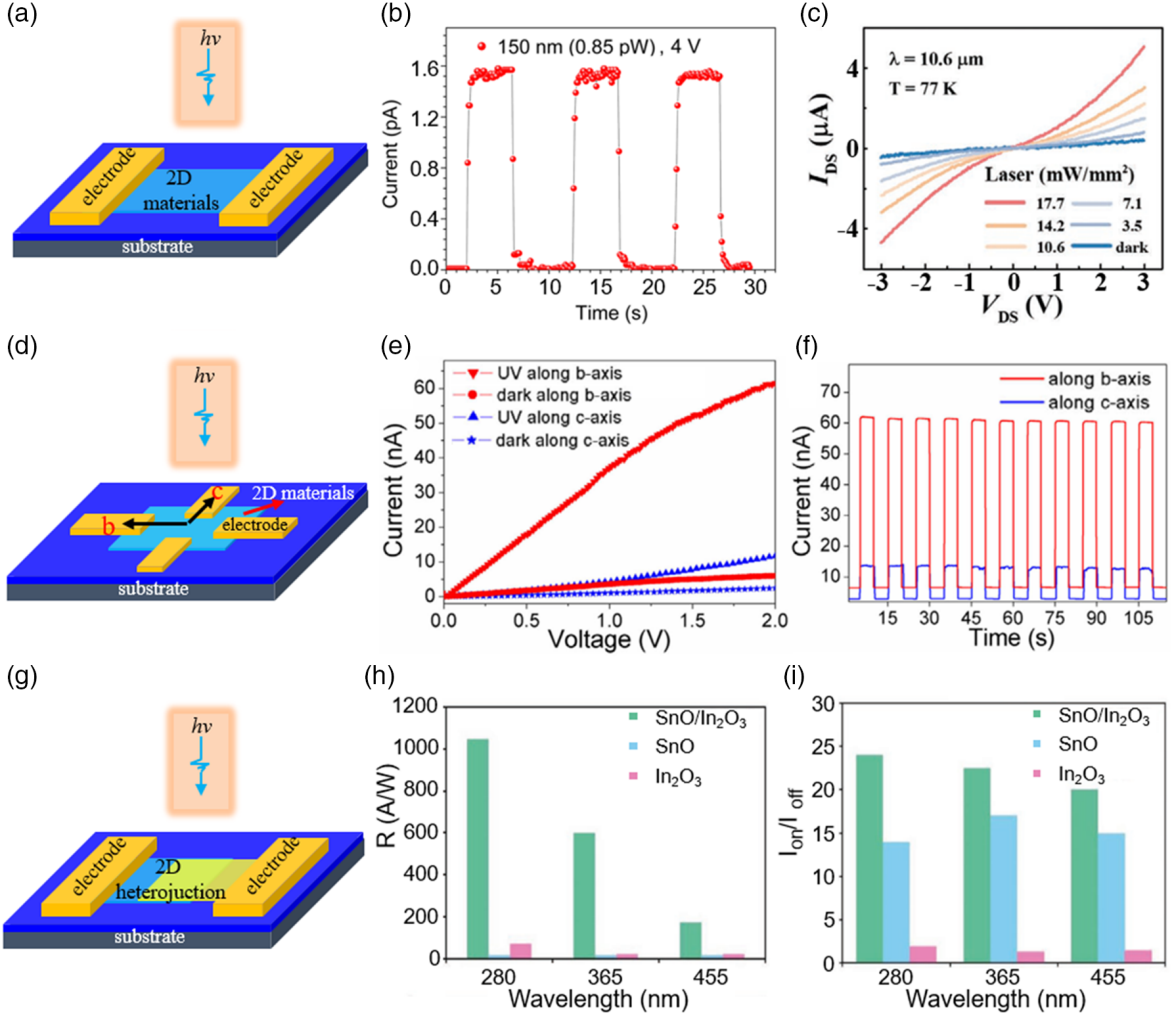
Photodetectors based on 2D oxides. a) Schematic diagram of photodetector. b) The time‐resolved current curves of the photodetector based on 2D MgO under 150 nm light with a power of 0.85 pW. Reproduced with permission.^[^ ^130^ ^]^ Copyright 2018, American Chemical Society. c) The *I–V* curve of the photodetector based on Fe_3_O_4_ under 10.6 μm light with different power densities. Reproduced with permission.^[^ ^19^ ^]^ Copyright 2020, Wiley‐VCH. d) Schematic diagram of the photodetector for anisotropic detection. e) The *I–V* curves of photodetectors based on α‐MoO_3_ in the dark and under illumination along the *b*‐ and *c*‐axis. Photocurrent–time curves of the α‐MoO_3_ photodetector along the *b*‐ and *c*‐axis. e, f) Reproduced with permission.^[^ ^27^ ^]^ Copyright 2018, IOP Publishing. g) Schematic diagram of photodetector based on heterojunction. h) Responsivity and i) current ratio of on/off of the photodetectors based on SnO/In_2_O_3_, SnO, and In_2_O_3_, respectively, under different wavelengths. h,i) Reproduced with permission.^[^ ^41^ ^]^ Copyright 2019, Wiley‐VCH.

The schematic diagram of the photodetector based on 2D materials is shown in **Figure** **11**a. Figure 11b shows time‐resolved current curves of the photodetector based on 2D MgO at vacuum ultraviolet light.^[^ ^130^ ^]^ Under the illumination of 150 nm light, the photodetector can even identify the extremely weak light signal of 0.85 pW, which can be attributed to the high charge collection efficiency of excited carriers within 2D MgO. This work provides a new way for developing next‐generation vacuum ultraviolet photodetectors.^[^ ^130^ ^]^ Recently, Yu et al.^[^ ^17^ ^]^ reported visible‐blind ultraviolet photodetectors based on ZnO nanosheets, which showed ultrahigh performance: the highest responsivity reached 2.0 × 10^4^ A W^−1^ and the detectivity was as high as 6.83 × 10^14^ Jones. Feng et al.^[^ ^18^ ^]^ built solar‐blind photodetectors based on 2D β‐Ga_2_O_3_, which showed not only high responsivity ≈3.3 A W^−1^ and detectivity ≈4.0 × 10^12^ Jones, but also high selectivity for the light wavelength. The photocurrent declines sharply when the incident wavelength exceeds 354 nm. Besides, some 2D oxides also exhibit great performance in visible and infrared detection. Yin et al.^[^ ^19^ ^]^ prepared ultrathin 2D Fe_3_O_4_ with a narrow bandgap for infrared detection and constructed photodetectors with high sensitivity due to the multimechanism synergistic effect of bolometric effect and photoconductive effect. The current–voltage (*I–V*) curves of the photodetector with different light power densities at 77 K are shown in Figure 11c. The optimal performance is obtained with laser wavelength of 10.6 μm: ultrahigh responsivity of 561.2 A W^−1^, external quantum efficiency of 6.6 × 10^3^%, and detectivity of 7.42 × 10^8 ^Jones. 2D oxides are also promising in the application of flexible photodetectors, thanks to their high flexibility. Yalagela et al.^[^ ^37^ ^]^ built the V_2_O_5_ photodetectors on cellulose paper. After 500 bending cycles, no notable change was observed in the responsivity values. Some of 2D materials have low symmetric crystal structure, resulting in in‐plane optical and electrical anisotropy. With research development, some 2D oxides with in‐plane anisotropy, such as α‐MoO_3_,^[^ ^27^ ^]^ have been found. Figure 11d shows the schematic diagram of anisotropy photodetector. Zhong et al.^[^ ^27^ ^]^ prepared α‐MoO_3_ and the crystal direction of the sample was determined by transmission electron microscopy (TEM). The two pairs of electrodes were fabricated along the *b*‐axis and *c*‐axis of α‐MoO_3_ respectively. The dark current, photocurrent, and the on/off ratio along *b‐*axis were 2.1, 4.3, and 1.8 times larger than along *c*‐axis, respectively (Figure 11e,f). The results mean that α‐MoO_3_ has great potential for application in anisotropic photoelectric detection. The construction of p–n heterojunction can improve the carrier transport efficiency of 2D materials interface.^[^ ^136^, ^137^ ^]^ As mentioned previously, both n‐ and p‐type semiconductor materials are abundant in 2D oxides, so it is convenient to construct p–n heterojunctions to obtain photodetectors with high performance. Alsaif and co‐workers^[^ ^41^ ^]^ constructed 2D SnO/In_2_O_3_ van der Waals heterostructure by printing the oxide skin of liquid metals.^[^ ^41^ ^]^ Figure 11h,i shows the photodetectors based on this heterostructure, which exhibited much better performance than the devices based on individual SnO or In_2_O_3_ in terms of responsivity and current ratio under light illuminations of 280, 365, and 455 nm.^[^ ^41^ ^]^ Wang et al. built heterojunction photodetectors based on p‐type 2D SnO and n‐type 2D MoS_2_. It showed excellent modulation effect by gate voltage.

To understand the research status of 2D oxide photodetectors systematically and comprehensively, some of the typical 2D oxides are used for photodetectors, as shown in **Table** **3**. In general, the main detection area is in the ultraviolet region as most of the 2D oxides have a wide bandgap. Some photodetectors based on 2D oxides present high performance.^[^ ^18^, ^24^, ^61^, ^138^, ^139^ ^]^. However, we also notice that most of the 2D oxides show low responsivity and detectivity, and the response times are close or more than 1000 ms, which means that there is still a long way for practical application. We analyze that the research in 2D oxides is still in its infancy, and the effective methods to produce high‐quality 2D oxide materials are still scarce.

**Table 3:** Summary of photodetectors based on 2D oxides

Wavelength [nm]	Materials	Strategies	Responsivity [A W^−1^]	Detectivity [Jones]	Response time [ms]	References
150	MgO	Conformal anneal	1.86	1.8 × 10^10^	–	[130]
250	Ga_2_O_3_	RF magnetron sputteringa)	6.62	–	1210/7140	[184]
254	ZnO	AILEb)	2 × 10^4^	6.83 × 10^14^	3970/5320	[17]
254	β‐Ga_2_O_3_	SOMc)	3.3	4.0 × 10^12^	30/60	[18]
254	β‐Ga_2_O_3_	MEd)	2.6 × 10^3^	9.7 × 10^13^	–	[185]
254	β‐Ga_2_O_3_	ME	1.8 × 10^5^	2.08 × 10^11^	670/1560	[186]
325	Cr_2_O_3_	CVDe)	0.62	–	–	[38]
330	ZnO	Electrochemical deposition	–	–	2.6 × 10^4^/1.1 × 10^4^	[139]
365	ZnO	SOM	12.64	5.81 × 10^15^	1.05 × 10^5^	[24]
365	Bi_2_O_3_	SOM	400	1.1 × 10^13^	0.07	[44]
532	MoO_3_/MoS_2_	CVD	5410	8.9 × 10^9^	100/100	[173]
532	MoO_2_/MoSe_2_	CVD	0.1	2.34 × 10^10^	550/720	[187]
10 600	Fe_3_O_4_	CVD	561.2	7.42 × 10^8^	–	[19]
254	α‐MoO_3_	Vapor–solid process	*R*_b_ 67.9f) *R*_c_ 6.1g)	–	45/25	[27]
280 356 455	SnO/In_2_O_3_	SOM	1407 600 173	5 × 10^9^ – –	–	[41]
254	MoSe_2_/MoO_*x*_	CVD	3.4	8.5 × 10^7^	400/500	[188]
532	MoO_3_/MoS_2_	CVD	5410	8.9 × 10^9^	100/100	[173]
532	MoSe_2_/MoO_2_	ME	< 0.1	–	430/1200	[187]

a) RF: radio frequency;

b) AILE: adaptive ionic layer epitaxy;

c) SOM: surface oxidation of metal;

d) ME: mechanical exfoliation;

e) CVD: chemical vapor deposition;

f) *R*_b_: responsivity along *b*‐axis;

g) *R*_c_: responsivity along *c*‐axis.

### 4.3 High‐*K* Dielectrc Layer and Encapsulation

**Figure 12:**
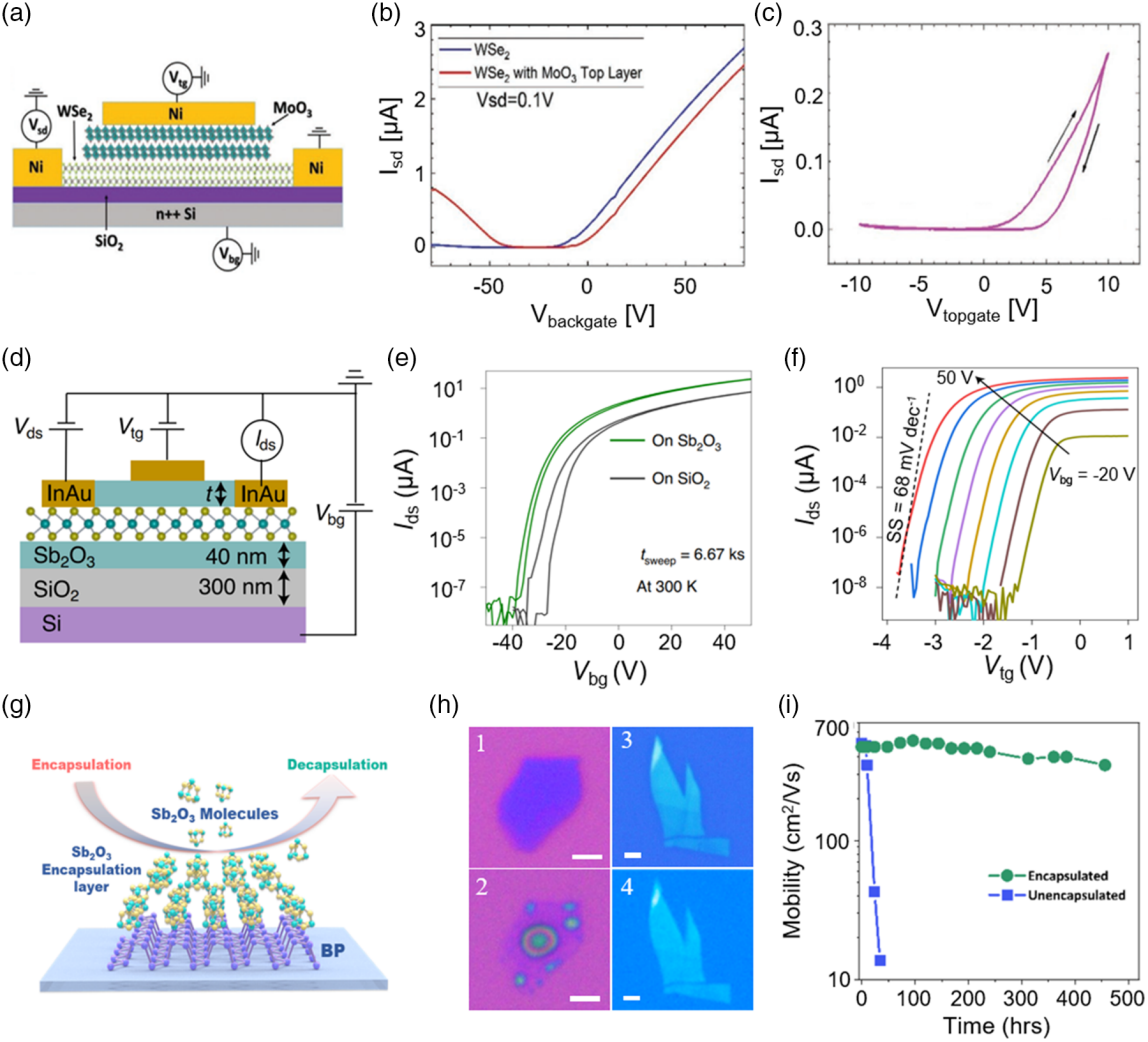
High‐*k* dielectric layer and encapsulation. a) Schematic diagram of the WSe_2_/MoO_3_ FET with top and back gate. b) Transfer characteristic curves of WSe_2_ FET and WSe_2_/MoO_3_ FET with back gate. c) Transfer characteristic curves of WSe_2_/MoO_3_ FET with top gate. a, b, c) Reproduced with permission.^[^ ^30^ ^]^ Copyright 2020, Wiley‐VCH. d) Schematic diagram of MoS_2_ FETs with Sb_2_O_3_ as top‐gate dielectric layer. e) Double‐sweep transfer characteristic curves of monolayer MoS_2_/Sb_2_O_3_ and MoS_2_/SiO_2_ FETs at 300 K with a low sweeping rate. f) Transfer characteristic curves of double‐gated FET with a sweeping top gate at various back‐gate voltages (SS). d–f) Reproduced with permission^[^ ^22^ ^]^ Copyright 2021, Springer Nature. g) Schematic diagram of Sb_2_O_3_ layer encapsulated onto and decapsulated from BP nanosheets. h) OM images of BP flakes (1,2 BP flake of fresh exfoliated after 1 day in ambient condition without encapsulated, respectively. 3,4 BP flake of fresh exfoliated after 60 day in ambient condition with encapsulated with 20 nm Sb_2_O_3_, respectively). i) The hole mobility of BP FETs versus ambient exposure time unencapsulated and encapsulated with Sb_2_O_3_. g–i) Reproduced with permission.^[^ ^144^ ^]^ Copyright 2021, Wiley‐VCH.

The performances of optoelectronic and electronic devices based on 2D materials are often affected in the air environment due to the poor environmental stability and high sensitivity. High‐*κ* dielectric materials such as HfO_2_ and Al_2_O_3_ were often used as encapsulation, gate dielectric, and passivation layer for protecting optoelectronic devices or enhancing their performance.^[^ ^140^, ^141^, ^142^, ^143^ ^]^ Considering the unique vdW nature of 2D materials, compatible dielectric materials are imperatively needed to increase coupling efficiency and realize seamless integration. Recently, Holler et al.^[^ ^30^ ^]^ reported that α‐MoO_3_ nanosheets obtained by mechanical exfoliation were used as dielectric. The measured results of α‐MoO_3_ have a high dielectric constant of ≈35 at low frequencies. Then they constructed WSe_2_/MoO_3_ FETs with back and top gates (the schematic is shown in **Figure** **12**a). Figure 12b shows the transfer curves of WSe_2_ FET before and after being covered by MoO_3_ layer. The FET based on WSe_2_ showed n‐type behavior in a typical back gate. After stacking the MoO_3_ layer, the device shifted toward p‐type behavior, indicating that holes were induced when the MoO_3_ layer was on top of WSe_2_. The transfer curves of WSe_2_/MoO_3_ FET with top gate are shown in Figure 12c. We noticed that the drain–source current can be controlled, which means that MoO_3_ nanosheets could be used as the dielectric layer. To achieve large‐area vdW dielectrics on 2D materials, Liu and co‐workers^[^ ^22^ ^]^ built MoS_2_ FETs after depositing Sb_2_O_3_ film. Due to the high molecular stability, Sb_2_O_3_ can be easily covered onto 2D materials via thermal evaporation deposition and contacted through vdW forces. The device structure is shown in Figure 12d. Figure 12e shows the transfer characteristic curves of monolayer MoS_2_ FETs with Sb_2_O_3_ and SiO_2_ used as sole dielectric, respectively. The monolayer MoS_2_ FETs with Sb_2_O_3_ dielectrics showed a smaller hysteresis window. The subthreshold slope (SS) reached a low value of 68 mV dec^−1^ at room temperature (Figure 12f). More importantly, compared with the MoS_2_/SiO_2_ FETs, MoS_2_/Sb_2_O_3_ FETs had higher mobility and on/off ratio (10^8^). Furthermore, Zhai's group^[^ ^144^ ^]^ also deposited Sb_2_O_3_ on 2D BP and realized wafer‐scale van der Waals encapsulation of 2D materials.^[^ ^144^ ^]^ It is well known that BP is an important 2D material with high performance, but its instability in air seriously limits its application in photoelectric and electronic. As an inorganic molecular crystal, Sb_2_O_3_ layer connects with the underlying 2D materials through vdW force due to its special structure which could avoid the structural damage to the protected materials. The schematic diagram of the Sb_2_O_3_ layer encapsulated onto and decapsulated from BP nanosheets is shown in Figure 12g. Figure 12h shows that the BP covered with Sb_2_O_3_ retained intact morphology after 60 days. In contrast, the BP flake without the encapsulation layer was drastically damaged after only 1 day of exposure in the air. The performance of BP FETs is strong evidence of the effect of encapsulation. Figure 12i presents that the hole mobility of BP FETs changed versus ambient exposure time. We can see that the FETs based on as‐exfoliated BP flakes without encapsulation deteriorate dramatically in mobility and decay rapidly to 0 within 2 days. However, the encapsulated BP FETs present high stability in the same environment; the mobility can be sustained for 19 days. In addition, the same results of applying the encapsulation approach to other 2D materials exhibit similar passivation effects.

### 4.4 Sensors

The special structure and superior optical and electrical properties of 2D materials make them promising as platforms and/or probes for developing sensitive sensors.^[^ ^145^, ^146^, ^147^, ^148^, ^149^, ^150^ ^]^ In recent years, the gas sensors and biosensors based on 2D oxides have been reported widely.^[^ ^151^, ^152^, ^153^, ^154^, ^155^, ^156^, ^157^, ^158^, ^159^ ^]^ In this section, we will introduce sensors based on 2D oxides electronic or optoelectronic devices.

#### 4.4.1 Gas Sensors

**Figure 13:**
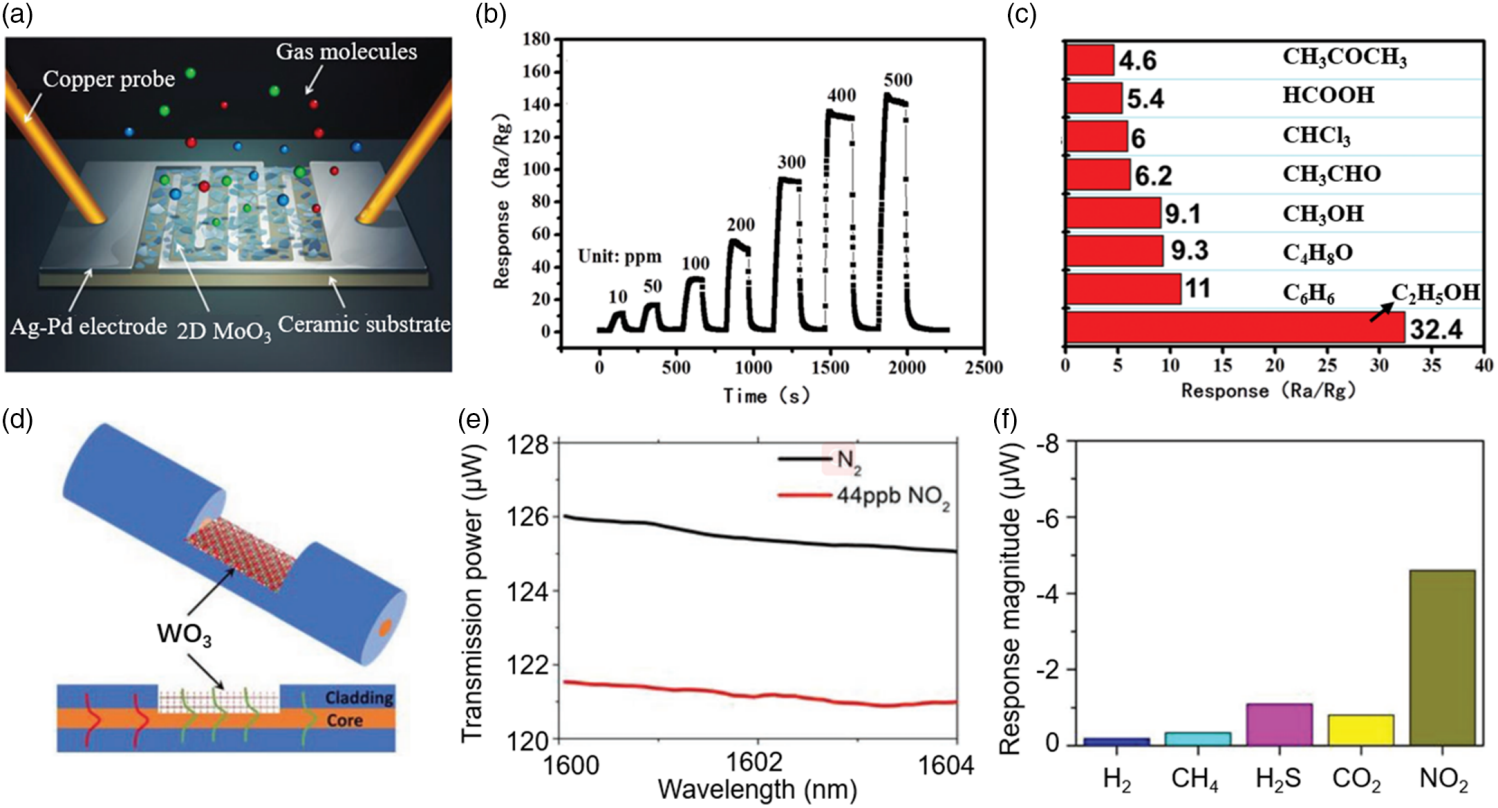
Sensors based on 2D oxides. a) Schematic illustration of the gas sensor based on 2D MoO_3_. b) Transient response and recovery curves of the 2D MoO_3_ sensor toward different alcohol vapor concentrations. c) 2D MoO_3_ sensor toward different 100 ppm VOCs. a–c) Reproduced with permission.^[^ ^161^ ^]^ Copyright 2016, The Royal Society of Chemistry. d) Schematics of the 2D WO_3_‐based fiber optics sensor. e) Transmission spectra of the 2D WO_3_‐coated D‐shape optical fiber with and without NO_2_ exposure. f) Selectivity measurement toward H_2_, CH_4_, H_2_S, CO_2_, and NO_2_. d–f) Reproduced with permission.^[^ ^162^ ^]^ Copyright 2019, Wiley‐VCH.

2D oxides have been fabricated to sensors to detect various gases including H_2_, NO_2_, H_2_S, CH_4_, methanol, ethanol, and so on.^[^ ^153^, ^156^, ^160^ ^]^ **Figure** **13**a shows the schematic illustration of the fabricated sensor based on 2D MoO_3_.^[^ ^161^ ^]^ The change in resistance of the device in ambient atmosphere and detecting gas atmosphere reflects the performance of the sensor. The transient response and recover curves of the sensor with different alcohol vapor concentrations are shown in Figure 13b. It shows that the sensitivity increases with alcohol vapor concentration. Under 100 ppm alcohol vapor atmosphere, the response time is 21 s and recovery time is 10 s. Figure 13c shows the sensitivity of the sensor made of MoO_3_ nanosheets toward 100 ppm volatile organic compounds (VOCs). We could notice clearly that the sensors have a relatively high sensitivity to alcohol vapor compared with other VOCs (about 3–7 times higher), indicating that MoO_3_ nanosheets have good selectivity to alcohol vapor.^[^ ^161^ ^]^ Yao et al.^[^ ^162^ ^]^ reported an ultrasensitive fiber optics gas sensor. The sensor shows a highly selective response to NO_2_ at low concentrations. Figure 13d illustrates the structure of fiber optics gas sensor based on 2D plasmonic WO_*x*_. The sensor follows a simple mechanism: electrical dipoles form on the surface when the gas molecules are absorbed on 2D plasmonic WO_*x*_, which causes charge transfer and the redistribution and polarization of free carriers within the 2D plasmonic system, and ultimately result in the variation of the absorption properties and the alternation of the evanescent field surrounding the fiber. From Figure 13e, we find that the transmission power of the sensor decreases upon the exposure of NO_2_ gas with a concentration of 44 part‐per‐billion (ppb). Figure 13f shows the gas selectivity of the sensor. The response magnitude for NO_2_ is much larger (about four times) than other common gases including H_2_, CH_4_, H_2_S, and CO_2_ at their low concentration.

#### 4.4.2 Biosensors

**Figure 14:**
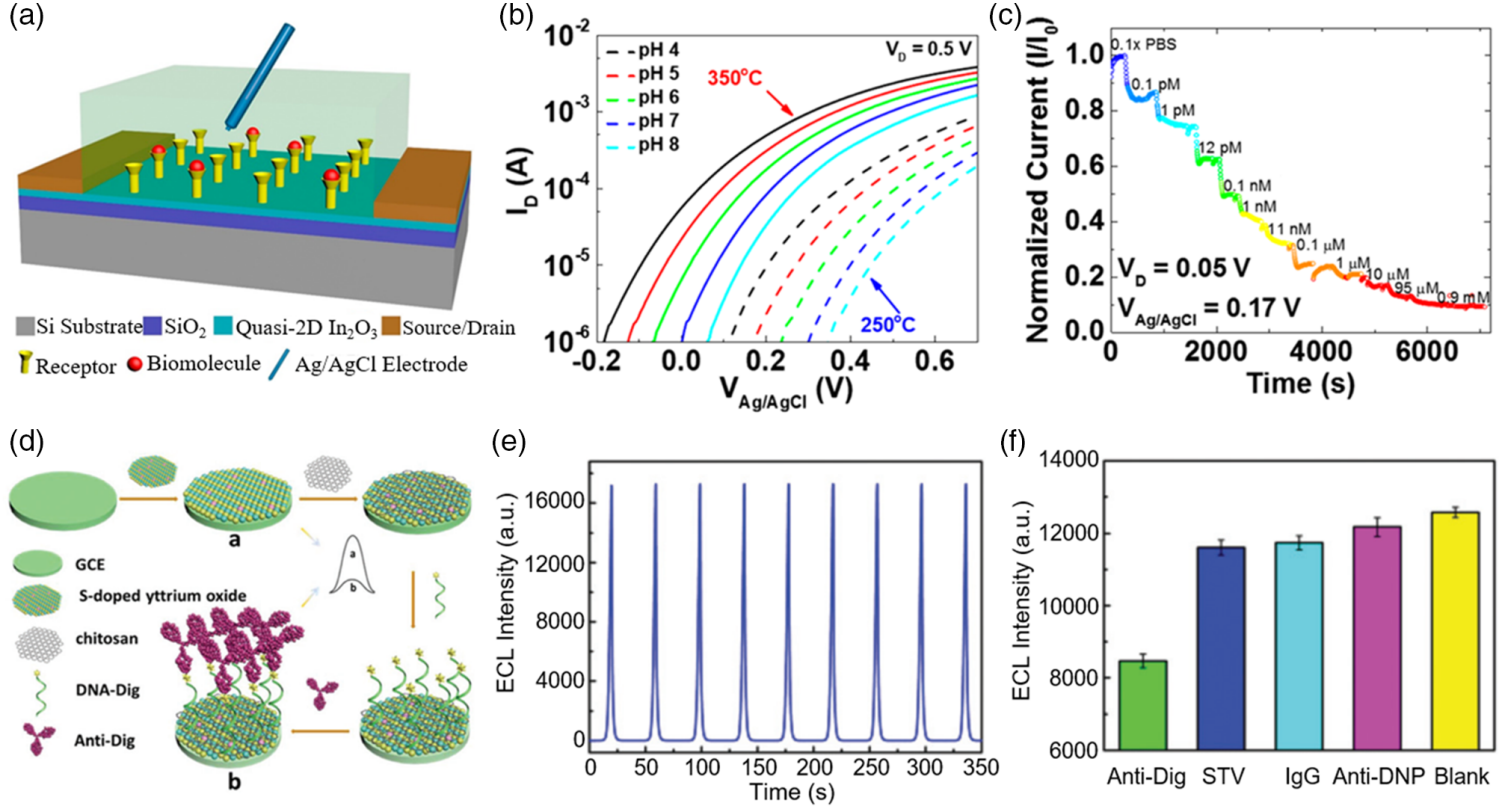
Biosensors based on 2D Oxides. a) Schematic representation of the 2D In_2_O_3_ bio‐FET and sensing scheme. b) Transfer characteristics after exposure to varying pH. c) Response for 2D In_2_O_3_ biosensor to various concentrations of glucose. a–c) Reproduced with permission.^[^ ^165^ ^]^ Copyright 2017, American Chemical Society. d) Schematic presentation of the ECL biosensor. e) Stability of the ECL response from the S‐doped Y_2_O_3_ nanosheets. f) ECL response of the biosensor towards different biomolecules and blanks. d–f) Reproduced with permission.^[^ ^174^ ^]^ Copyright 2018, The Royal Society of Chemistry.

Some 2D oxides are used to build transistor‐based biosensors (Bio‐FETs).^[^ ^163^, ^164^ ^]^ Chen et al.^[^ ^165^ ^]^ built quasi‐2D In_2_O_3_ Bio‐FETs, achieving pH sensor with detection limits as low as 0.0005 and glucose sensor with detection limits less than 7 fM. **Figure** **14**a shows the schematic representation of the Bio‐FET based on 2D In_2_O_3_ and sensing scheme.^[^ ^165^ ^]^ Reference electrode, biomolecule receptors, and electrolyte replaced the traditional gate and dielectric structure of FETs. The changes in the electrical properties of Bio‐FETs can be used to reflect the changes in bioanalyte. The transfer curves of the channel‐sensing surfaces exposed to buffer solution with different pH values are presented in Figure 14b. Under the same test conductions, the turn‐on voltage (*V*_on_) and drain current (*I*_D_) show a linear change with pH values. The bio‐FETs also show high sensitivity for the response of glucose. Figure 14c demonstrates the continuous monitoring of sensing signal responses as a function of glucose concentration and the limit of detection below 7 fM. The demonstrated show that the high sensitivity of bio‐FETs may be attributed to the carriers in 2D semiconductors that are confined within the channel, and the electronic perturbation at the surface will lead to a remarkable effect on the resistance of the transistors. The electrochemiluminescence (ECL) properties of materials have been used to construct biosensors. Guo and co‐workers designed an ECL biosensor for detecting anti‐Dig antibodies, as shown in Figure 14d. S‐doped Y_2_O_3_ exhibits good ECL performance, and Dig molecules‐linked DNA can grapple anti‐Dig antibodies, which has the ability of moleculeprotein recognition. The consecutive high ECL emission of the S‐doped Y_2_O_3_ nanosheets is shown in Figure 14e, from which we can see that the nanosheets have long‐standing ECL stability. Figure 14f exhibits the ECL response of the biosensor toward blank and different analytes that include anti‐Dig antibodies (10 nM), streptavidin (STV) (100 nM), human immunoglobulin G (IgG) (100 nM), and anti‐dinitrophenol (anti‐DNP) (100 nM) under the same experimental conditions. Despite the lower concentration, anti‐Dig has more pronounced inhibition for ECL intensity. These results suggest that the biosensor presents excellent selectivity for the detecting of anti‐Dig antibodies.

### 4.5 Other Applications

**Figure 15:**
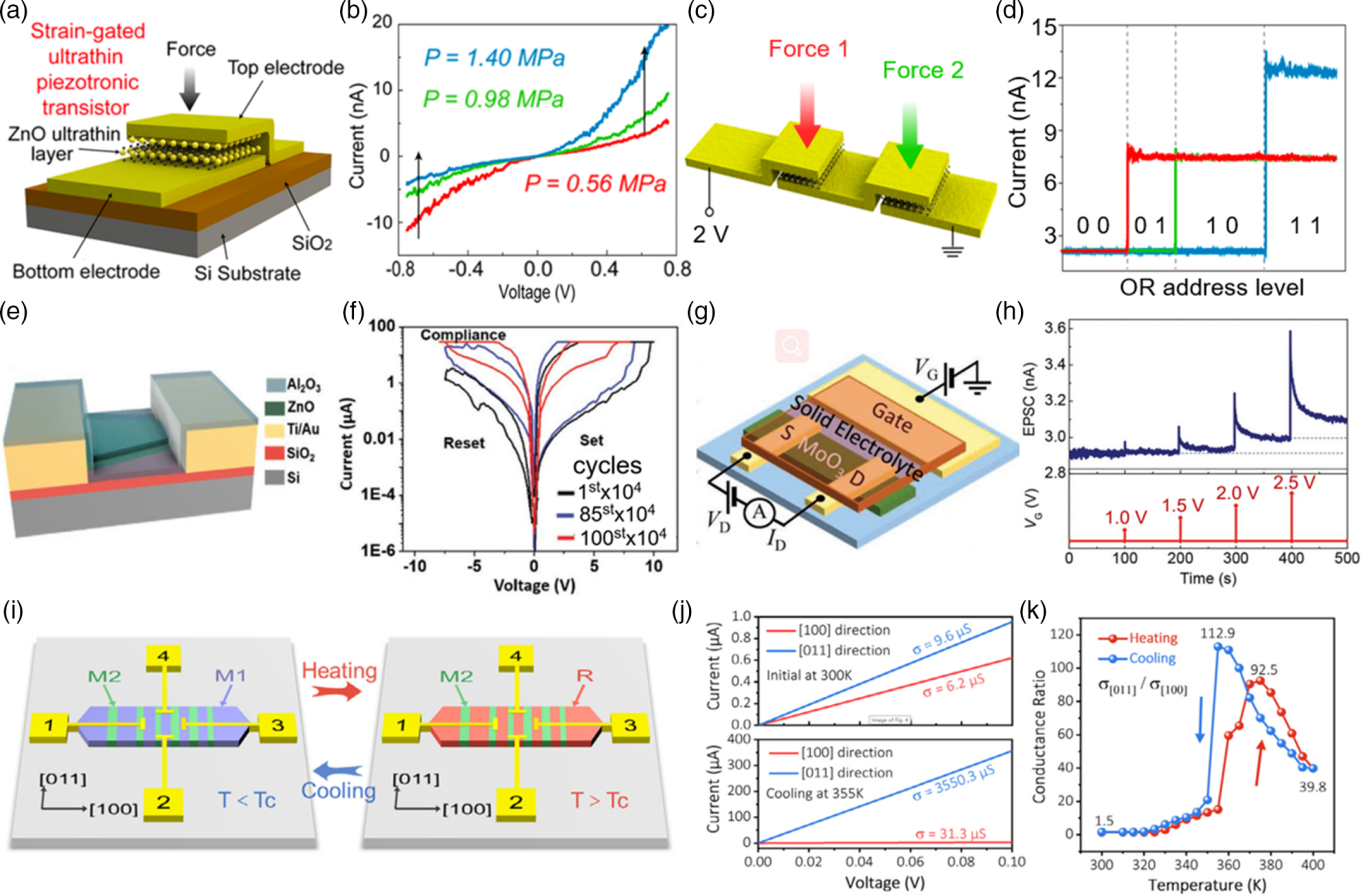
Other electronic and optoelectronic applications of 2D oxides. a) Schematic diagram of the ZnO piezotronic transistor with two‐terminal configuration. b) *I–V* curves of ZnO piezotronic transistors under different pressures. c) Stress‐gated OR logic gates using a series of ZnO piezotronic transistor. d) Red line corresponding to OR gate with the force 1 on and the measured output currents for “01” state. Green line corresponding to OR gate with the force 2 on and the measured output currents for “10” state. Blue line corresponding to OR gate with the both force 1 and force 2 on and the measured output currents for “11” state. a–d) Reproduced with permission.^[^ ^23^ ^]^ Copyright 2018, American Chemical Society. e) Schematic of a ZnO/Al_2_O_3_ heterolayered nanosheet memristor. f) Three *I–V* curves of the ZnO nanosheet memristor at the 1 × 10^4^, 85 × 10^4^, and 100 × 10^4^th cycle. e,f) Reproduced with permission.^[^ ^26^ ^]^ Copyright 2020, Wiley‐VCH. g) Schematic diagram of the three‐terminal electrochremical transistors. h) Excitatory postsynaptic current stimulated by a series of gate voltage pulses with the same duration time (10 ms) and different amplitudes (1.0, 1.5, 2.0, and 2.5 V). g, h) Reproduced with permission.^[^ ^166^ ^]^ Copyright 2018, Wiley‐VCH. i) Design of the in‐plane electrical anisotropy measurement by cross‐type electrode pairs. j) The initial *I–V* curves along the [011] and the [100] directions at 300 K and the maximum current difference curves along the [011] axis and the [100] axis during the cooling process at 355 K. k) The evolution of the conductance ratio in a cycle covering the temperature range from 300 to 400 K. i–k) Reproduced with permission.^[^ ^28^ ^]^ Copyright 2021, KeAi Publishing.

2D oxides have potential for applications in many electronic and optoelectronic devices. Although most of them are still in their infancy, related studies have been reported in recent years. Wang et al.^[^ ^23^ ^]^ demonstrated ultrathin ZnO piezotronic transistors with the channel length of only 2 nm. The structure of the device is shown in **Figure** **15**a. With the increase in stress, the *I–V* curves of the piezotronic transistor showed asymmetric modulation (Figure 15b): the current significantly increased under forward bias and gradually decreased under reverse bias, which indicated that the charge carrier transport was dominated by piezotronic effect. In addition, logic gates could be constructed using a series of ZnO piezotronic transistors (Figure 15c,d). The study showed the potential application of ultrathin ZnO piezotronic transistors in next‐generation electronics. Yin and co‐workers^[^ ^26^ ^]^ reported the memristor based on the amorphous–crystalline 2D oxide heterostructure and the conduction mechanism is that the crystalline ZnO nanosheets provide a 2D host for oxygen vacancies, while the amorphous Al_2_O_3_ facilitates the generation and stabilization of oxygen vacancies. Figure 15e shows the schematic diagram of memristive device based on ZnO/Al_2_O_3_ heterolayered nanosheet. The device exhibited excellent memristive behavior: set/reset cycles up to 1 × 10^6^ times (Figure 15f). In addition, a high carrier mobility of 2400 cm^2^ V^−1^ s^−1^ was obtained in the low‐resistance state. Electronic synaptic devices are one important part of neuromorphic computational systems. As a research hotspot, many 2D materials have been applied in this field. Yang et al.^[^ ^166^ ^]^ reported an all‐solid‐state electrochemical transistor (ECT) based on 2D α‐MoO_3_ nanosheets, as shown in Figure 15g. Nonvolatile conductance modulation was achieved in a low‐conductance regime by reversible intercalation of Li^+^ into the α‐MoO_3_ lattice. Figure 15h shows a series of voltage pulses with duration time of 10 ms and 1.0, 1.5, and 2.0 V amplitudes applied on the gate as external action potential, respectively. The corresponding excitatory postsynaptic current (EPSC) was measured at a drain‐source voltage of 50 mV. Behaviors similar to biological synapses was observed: with the applying of voltage pulses, the EPSC reached peak value at the end of the pulse and then decayed back. The peak value of EPSC increased with the amplitude of the voltage pulse. This work proved the potential of 2D oxide ECT devices in neuromorphic computational networks. In‐plane anisotropy 2D materials have attracted extensive attention due to their difference of in‐plane optical and electrical properties in different crystal orientations.^[^ ^83^, ^167^, ^168^ ^]^ Ran et al.^[^ ^28^ ^]^ presented a periodic phase engineering strategy to enhance the in‐plane anisotropy of VO_2_ nanosheets. By introducing alternant monoclinic and rutile phases in the VO_2_ nanosheet, an ultrahigh in‐plane anisotropic electrical conductivity of 113 was obtained. The device for the measurement of in‐plane anisotropy was designed and is shown in Figure 15i. M1 and M2 are two different types of monoclinic phases VO_2_, and R phase VO_2_ is metallic and transformed from M1 at 335–340 K. Two pairs of electrodes of 1–3 and 2–4 were fabricated along the [100]_M1_ and [011]_M2_ of the VO_2_ nanosheet, respectively. The *I–V* curves along the [100] and the [011] directions at 300 K and the cooling process at 355 K are shown in Figure 15j. Compared with original data, the in‐plane anisotropic electrical conductivity increased significantly when temperature cooled at 355 K. Figure 15k exhibits the evolution of the conductance ratio in a cycle with the temperature range from 300 to 400 K. The results mean that the strategy to enhance the in‐plane anisotropy of VO_2_ nanosheets is effective, and the process of phase change is reversible. The work may provide opportunities for polarization‐dependent electronic and optoelectronic devices. We believe that with further studies, 2D oxides will present more novel phenomena and broader applications in electronics and optoelectronics.

In general, great progress has been made in the electronic and optoelectronic devices of 2D oxides, but there is still a gap compared with graphene, TMDs, and BP. For example, most of the researches on 2D oxide FETs is based on simple device configuration, while there are few reports on the FETs with complex structure. The photodetectors based on 2D oxides show excellent performance in UV detection but are poorly in the visible and infrared range. Significantly, with the development of the research, 2D oxides show more applications, such as a high‐*κ* dielectric layer, sensors, electronic synaptic devices, and neuromorphic computational networks. Although most of the researches are still in their infancy, the results are encouraging.

## 5 Summary and Prospect

In this review, we summarized the recent progress of 2D oxides, mainly focusing on the synthesis methods and potential application in electronics and optoelectronics. Based on the elements, the reported 2D oxides are classified into TMOs, MMOs, and SMOs. We introduced the structures and basic optical and electronic properties of some typical 2D oxides. After studies in recent years, various synthesis methods have emerged for the preparation of 2D oxides such as mechanical exfoliation, LPS, vapor deposition, and SOM. Among them, the mechanical exfoliation method could provide the highest quality of 2D oxides, but the method is only effective for the layered materials with weak interlayer forces. Moreover, the size and thickness of the obtained samples are difficult to control and the process is time‐consuming. It is generally suitable for fundamental research. At present, despite some problems, CVD and SOM are relatively more potential methods to achieve the preparation of 2D oxides with high quality and large scale. The application of reported 2D oxides in electronics and optoelectronics mainly focuses on the FETs and photodetectors. Considering that p‐type semiconductors with high mobility and excellent stability are scarce in common 2D materials, 2D oxides have great potential in the applications of FETs. 2D oxides are widely used in the field of photodetectors with the detection range from deep ultraviolet to midinfrared. For the detection in the ultraviolet region, some 2D oxides have advantages of high sensitivity and outstanding selectivity. In addition, 2D oxides have gradually shown potential in piezoelectric, information storage, memory, and artificial synapses.

Although great progress has been made in recent years, the research in 2D oxides is still at its infancy and there are several challenges that need to be overcome for their further applications. Here are some main challenges and reasonable perspectives. 1) Some 2D oxides with excellent properties are still at the theoretical research stage.^[^ ^169^, ^170^ ^]^ For example, Can et al.^[^ ^169^ ^]^ predicted that twisted double‐layer copper oxides will exhibit high‐temperature topological superconductivity, and the structure can be realized by mechanical exfoliation. So it is necessary to experimentally synthesize these materials and study their applications in electronics and optoelectronics. 2) There is lack of efficient methods for the preparation of high‐quality and large‐scale 2D Oxides. At present, the method of SOM could realize the preparation of 2D oxides with large scale, but it is difficult to ensure reliable continuity. CVD is considered as an ideal method for preparing 2D materials with high quality and large scale.^[^ ^7^ ^]^ However, the size of reported 2D oxides prepared by the CVD method is relatively small. Recently, by designing the substrate, researchers have successively realized the fabrication of wafer‐scale single‐crystal h‐BN,^[^ ^116^ ^]^ MoS_2_^[^ ^115^ ^]^ and WS_2_.^[^ ^171^ ^]^ This strategy probably inspires the development in the growth of 2D oxides. 3) Some p‐type 2D oxides exhibit high mobility and air stability.^[^ ^20^, ^21^ ^]^ For example, the hole mobility of few‐layer h‐TiO_2_ reaches 950 cm^2^ V^−1^ s^−1^ at room temperature, which can be good supplementary to the mainstream 2D materials.^[^ ^20^ ^]^ However, the research on these materials is still at the beginning, and only simple single FET devices have been constructed. The studies of p‐type 2D oxides in electronics and optoelectronics need to be further developed, such as building low‐energy transistors, multifunction devices, and so on. 4) At present, 2D Oxides are mainly used for FETs and photodetectors. Although the piezoelectric, memory, artificial synapses, and other fields are also involved, the current researches are still at the preliminary stage and there are only a few reports about them. So, great efforts are still required to extend the applications of 2D oxides beyond FETs and photodetectors in the fields of electronics and optoelectronics. 5) To adapt to the trend of devices becoming miniaturized, integrated, and wearable in the future, it is necessary to develop reliable techniques for large‐scale transfer of 2D oxides to flexible substrates or in situ growth of 2D oxides on flexible substrates. In addition, the integration of devices based on 2D oxides on flexible substrates needs further exploration to accelerate their application in flexible electronics. 6) Heterojunction plays an important role in electronics and optoelectronics.^[^ ^172^ ^]^ So the heterostructures of 2D oxides and other 2D materials (such as graphene, TMDs, and BP) can be constructed to improve the performance of the devices. For example, the photodetector based on the heterostructure of p‐type β‐TeO_2_ and n‐type Bi_2_O_2_Se may exhibit fast response speeds due to their high carrier mobility which will induce the separation of photogenerated carriers. In addition, building heterojunctions can broaden the application of 2D oxides in electronics and optoelectronics, such as light‐emitting diodes, logic circuits, rectifiers, and inverters.

## Conflict of Interest

The authors declare no conflict of interest.
